# Novel biallelic variants expand the *SLC5A6*-related phenotypic spectrum

**DOI:** 10.1038/s41431-021-01033-2

**Published:** 2022-01-11

**Authors:** Tess Holling, Sheela Nampoothiri, Bedirhan Tarhan, Pauline E. Schneeberger, Kollencheri Puthenveettil Vinayan, Dhanya Yesodharan, Arun Grace Roy, Periyasamy Radhakrishnan, Malik Alawi, Lindsay Rhodes, Katta Mohan Girisha, Peter B. Kang, Kerstin Kutsche

**Affiliations:** 1 Institute of Human Genetics, University Medical Center Hamburg-Eppendorf, 20246 Hamburg, Germany; 2 Department of Pediatric Genetics, Amrita Institute of Medical Sciences & Research Centre, Cochin, 682041 Kerala India; 3 Division of Pediatric Neurology, Department of Pediatrics, University of Florida College of Medicine, Gainesville, FL 32610 USA; 4 Department of Pediatric Neurology, Amrita Institute of Medical Sciences & Research Centre, Cochin, 682041 Kerala India; 5 Department of Neurology, Amrita Institute of Medical Sciences & Research Centre, Cochin, 682041 Kerala India; 6 Suma Genomics Pvt. Ltd, Manipal Universal Technology Business Incubator (MUTBI), Manipal, 576104 India; 7 Bioinformatics Core, University Medical Center Hamburg-Eppendorf, 20246 Hamburg, Germany; 8 GeneDx, Gaithersburg, MD 20877 USA; 9 Department of Medical Genetics, Kasturba Medical College, Manipal, 576104 India; 10 Paul and Sheila Wellstone Muscular Dystrophy Center, University of Minnesota Medical School, Minneapolis, MN 55455 USA; 11 Department of Neurology, University of Minnesota Medical School, Minneapolis, MN 55455 USA; 12 Institute for Translational Neuroscience, University of Minnesota Medical School, Minneapolis, MN 55455 USA; 13 Present Address: Amedes MVZ Wagnerstibbe für Laboratoriumsmedizin, Hämostaseologie, Humangenetik und Mikrobiologie Hannover, 30159 Hannover, Germany

**Keywords:** Peripheral neuropathies, Genetics research

## Abstract

The sodium (Na^+^):multivitamin transporter (SMVT), encoded by *SLC5A6*, belongs to the sodium:solute symporter family and is required for the Na^+^-dependent uptake of biotin (vitamin B7), pantothenic acid (vitamin B5), the vitamin-like substance α-lipoic acid, and iodide. Compound heterozygous *SLC5A6* variants have been reported in individuals with variable multisystemic disorder, including failure to thrive, developmental delay, seizures, cerebral palsy, brain atrophy, gastrointestinal problems, immunodeficiency, and/or osteopenia. We expand the phenotypic spectrum associated with biallelic *SLC5A6* variants affecting function by reporting five individuals from three families with motor neuropathies. We identified the homozygous variant c.1285 A > G [p.(Ser429Gly)] in three affected siblings and a simplex patient and the maternally inherited c.280 C > T [p.(Arg94*)] variant and the paternally inherited c.485 A > G [p.(Tyr162Cys)] variant in the simplex patient of the third family. Both missense variants were predicted to affect function by in silico tools. 3D homology modeling of the human SMVT revealed 13 transmembrane helices (TMs) and Tyr162 and Ser429 to be located at the cytoplasmic facing region of TM4 and within TM11, respectively. The *SLC5A6* missense variants p.(Tyr162Cys) and p.(Ser429Gly) did not affect plasma membrane localization of the ectopically expressed multivitamin transporter suggesting reduced but not abolished function, such as lower catalytic activity. Targeted therapeutic intervention yielded clinical improvement in four of the five patients. Early molecular diagnosis by exome sequencing is essential for timely replacement therapy in affected individuals.

## Introduction

The sodium (Na^+^):multivitamin transporter (SMVT) is a member of the sodium:solute symporter (SSS) family, a group of membrane proteins mediating the transport of the respective solute(s) by using the sodium gradient. SMVT is encoded by *SLC5A6* and forms together with other members of the SSS family the solute carrier family 5 (SLC5) [1]. SMVT is required for the Na^+^-dependent uptake of the water-soluble vitamins pantothenic acid (vitamin B5) and biotin (vitamin B7), the vitamin-like substance α-lipoic acid, and iodide [2–5]. The organic substrates of the SMVT are essential for human health [2]. Biotin is a coenzyme for several carboxylases, is involved in various metabolic reactions, and has a role in gene expression regulation, cell proliferation and survival [6]. Pantothenic acid is a component of coenzyme A and fatty acid synthase necessary for energy production and hormone synthesis [7]. α-lipoic acid serves as a cofactor in redox reactions important for mitochondrial energy production and amino acid metabolism [8]. SMVT’s role in iodide transport and homeostasis is unknown up to date [2, 3].

Because SMVT substrates have versatile metabolic functions, deficiency of biotin, pantothenic acid and/or α-lipoic acid may lead to a broad range of clinical features in affected individuals. For example, biotin-dependent inherited disorders are holocarboxylase synthetase and biotinidase deficiency caused by biallelic variants in *HLCS* and *BTD*, respectively. Patients show a spectrum of clinical features including alopecia, skin rash, difficulties in breathing, hypotonia, seizures, developmental delay, feeding problems, vomiting, organic aciduria, and metabolic acidosis [6]. Thus, it is not surprising that compound heterozygous variants in *SLC5A6* are associated with variable multisystemic manifestations in four patients reported to date. A 15-month-old patient with a c.280 C > T [p.(Arg94*)] variant in trans with the c.368 G > T [p.(Arg123Leu)] variant had failure to thrive, microcephaly, developmental delay, spastic cerebral palsy, brain atrophy, immunodeficiency, and osteopenia [9]. A 3-year-old girl with compound heterozygous *SLC5A6* p.(Val141Alafs*34) and p.(Gln622Argfs*51) variants had failure to thrive and delayed motor development in infancy. At 17 months, she developed a severe metabolic crisis with hypoglycemia during gastroenteritis requiring resuscitation [10]. Two siblings with c.422_423del and c.1199 G > C/p.(Arg400Thr) biallelic variants had normal early development, followed by neurological regression and truncal ataxia with dyskinetic movements at the age of 12 and 14 months. One sibling developed seizures, cyclic vomiting and peripheral neuropathy at 7 years. The other sibling died at 2 years 7 months due to acute gastrointestinal hemorrhage [11]. Targeted vitamin replacement therapy resulted in clinical improvement [9–11]. Here we report five individuals from three families, including three siblings and two singletons, with biallelic variants in *SLC5A6* and motor neuropathies.

## Subjects and methods

### Whole-exome sequencing and variant filtering

Whole-exome sequencing (WES) was performed in patient 1–2, patient 2–1 and parents, and patient 3–1.

For detailed descriptions of the applied sequencing techniques, see the online Supplementary Information.

*SLC5A6* variants were described according to the GenBank reference sequences NM_021095.4 and NP_066918.4. The *SLC5A6* variants were submitted to the LOVD database (https://databases.lovd.nl/shared/genes/SLC5A6), with LOVD Variant IDs 0000784069, 0000784070, 0000784071, 0000784072, and 0000813612.

### Protein structure homology modeling

For detailed description, see the online Supplementary Information.

### Expression constructs and mutagenesis

Human *SLC5A6* coding region was PCR-amplified from fibroblast-derived cDNA. The PCR product was cloned into pENTR/D-TOPO vector (Thermo Fisher Scientific) according to the manufacturer’s protocol. The variants c.368 G > T [p.(Arg123Leu)], c.485 A > G [p.(Tyr162Cys)], and c.1285 A > G [p.(Ser429Gly)] were introduced in the *SLC5A6* coding region using the In-Fusion HD Cloning Kit (Takara) according to the manufacturer’s protocol for mutagenesis. The SLC5A6 wildtype and the three mutant constructs were used for transferring the coding region into the mammalian expression vector pEGFP-N3 using the In-Fusion HD Cloning Kit (Takara) according to the manufacturer’s protocol for cloning. All constructs were sequenced for integrity.

### Immunocytochemistry and confocal microscopy

A total of 150,000 HeLa cells were cultivated in Dulbecco’s modified Eagle medium (DMEM; Thermo Fisher Scientific) supplemented with 10% fetal bovine serum (FBS; GE Healthcare) and penicillin-streptomycin (100 U/ml and 100 µg/ml, respectively; Thermo Fisher Scientific) on glass coverslips in 6-well plates. Cells were transiently transfected with C-terminally EGFP-tagged SLC5A6 expression constructs (wildtype and mutants) using jetOPTIMUS (Polyplus Transfection) with a DNA (µg):jetOPTIMUS (µL) ratio of 1:1 and cultivated for 48 h before fixing cells in 4% paraformaldehyde in phosphate-buffered saline (PBS). After extensive washing with PBS, cells were embedded in mounting solution (ProLong Diamond Antifade Mountant; Thermo Fisher Scientific). Cells were analyzed and images were acquired with the Leica TCS SP8 X confocal microscope.

## Results

### Clinical summaries

Patient 1–1 is the first child born full term to healthy, non-consanguineous parents (Table 1 and Supplementary Table 1). Early development was normal. She underwent a right inguinal herniorrhaphy at 18 months. At 14 years, she presented with a 6-month history of difficulty holding a pen and fine motor difficulties in the right hand. Her left hand function was normal. She denied difficulty in walking, climbing stairs or rising from a squatting position. The initial physical exam was remarkable for distal right upper extremity weakness, along with wasting of the thenar and hypothenar eminences and interossei bilaterally. She had difficulty opposing her thumb to the 4th and 5th fingers, but handgrip was good. Nerve conduction studies (NCS) revealed signs of focal demyelination in the right upper extremity (Supplementary Table 2). Needle electromyography (EMG) was not performed.

**Table 1:** Main clinical features in five patients with biallelic *SLC5A6* variants.

		Patient 1–1	Patient 1–2	Patient 1–3	Patient 2–1	Patient 3–1
Variant	NM_021095.4 NP_066918.4	c.1285 A > G p.(Ser429Gly) Homozygous	c.1285 A > G p.(Ser429Gly) Homozygous	c.1285 A > G p.(Ser429Gly) Homozygous	c.280 C > T p.(Arg94*) c.485 A > G p.(Tyr162Cys)	c.1285 A > G p.(Ser429Gly) Homozygous
Demographics	Age of onset	13 y 6 m	10 y	6 y 9 m	8 y 5 m	8 y
	Age of diagnosis	14 y	11 y 9 m	6 y 9 m	9 y 3 m	13 y
	Sex	Female	Female	Male	Female	Female
Measurements	Age	14 y	11 y 9 m	6 y 9 m	9 y 3 m	13 y
	Weight (centile) (z)	34 kg (<1st) (−2.44)	26.3 kg (1st) (−2.55)	19.5 kg (14th) (−1.06)	23.5 kg (7th) (−1.44)	48 kg (59th) (−0.23)
	Height (centile) (z)	151 cm (8th) (−1.43)	133.5 cm (2st) (−2.12)	119 cm (41st) (−0.22)	131.7 cm (35th) (−0.39)	148 cm (82th) (−1.31)
	OFC (centile) (z)	54 cm (37th) (−0.33)	53.5 cm (54th) (+0.10)	52 cm (49th) (−0.02)	ND	ND
Variant Neuromuscular Exam Findings at Diagnosis	Neurological development	Normal	Normal	Normal	Normal	Normal
	Muscle Bulk	Atrophy of thenar and hypothenar eminence (more prominent on the right hand); bilateral atrophy of interosseous muscles	Atrophy of thenar and hypothenar eminence (more prominent on the right hand); bilateral atrophy of interosseous muscles	Normal	Muscle bulk reduced throughout; bilateral atrophy of thenar eminence and interosseous muscle	Atrophy of thenar and hypothenar eminence (more prominent on the right hand and thenar eminence) and interosseous muscles
	Muscle strength					
	Neck flexion/ extension	5/5	5/5	5/5	5/5	5/5
	Upper extremity	Difficulty in pincer grasp and fine movements of fingers and opposition of thumb with 4th and 5th fingers; contracture of bilateral 4th and 5th toes	Distal weakness of hands; difficulty in pincer grasp and opposition of thumb with 4th and 5th fingers; contracture of bilateral thumbs and right index finger	Mild hand grip weakness	Deltoids 3 Triceps 5 Biceps 4 Wrist Flexion & extension 4/5 Extensor digitorum communis 4 First dorsal interosseous 2 Abductor digitiminimi 2 Abductor pollicis brevis 0 Grip 4	Shoulder, elbow, and wrist flexion/extension 5/5 Distal weakness of distal upper extremity including palmar and dorsal interossei, abductor pollicis, adductor pollicis, opponens pollicis, and flexor pollicis brevis. Lumbrical 5 Grip 4+
	Lower extremity	No proximal muscle weakness of lower limbs	Bilateral weak ankle dorsiflexion and bilateral 4th and 5th toes; no proximal muscle weakness of lower limbs	Normal power in muscles of lower limb	Gluteus medius 5 Gluteus maximus 5 Iliopsoas 4 Quadriceps 5 Hamstrings 3 Tibialis anterior 3 Tibialis posterior 2 Peroneus longus/brevis 5 Extensor hallucis longus 3 Gastrocnemius 4	b/l hip flexion 4 (left > right) Extension 5 Adduction 5 Abduction 5 Knee flexion 4− (left > right) Knee extension 5 Ankle dorsiflexion 4 Plantar flexion 5 (Eversion weaker than Inversion on the right Inversion weaker than eversion on the left)
	Reflexes	Sluggish in upper and lower limbs	Sluggish in upper and lower limbs	Deep tendon reflexes in upper and lower limbs are normal	2+ in upper extremity; absent in lower extremity; down going plantar response B/L	Brisk in upper extremity, sluggish in lower extremity and absent ankle reflexes B/L
	Sensation	Normal	Normal	Normal	Normal	Normal
	Gait	Normal	Normal	Normal	Unsteady with smaller steps; unable to perform toe/heel walking and tandem walking	Ataxic gait with minimal waddling; unable to perform tandem walking. Dysmetria, dysdiadokokinesia + (more on left side)
Diagnostic Workup	Needle EMG	ND	Chronic denervation	ND	The left anterior tibialis and medial gastrocnemius muscles displayed polyphasic motor units with mildly prolonged durations and the left first dorsal interosseous displayed mildly prolonged motor unit durations	Initial study at 11 y showed denervation-reinnervation pattern at the right FDI, with normal patterns at the right TA, deltoid, and brachioradialis. Follow-up study at 13 y showed discrete recruitment patterns at all muscles tested
	Muscle/nerve biopsy	ND	Muscle biopsy: neurogenic pattern	ND	Muscle biopsy: neurogenic pattern with scattered small atrophic angulated myofibers of both types and fiber type grouping. Sural nerve biopsy: normal	ND

Motor power values for neck, upper extremity, and lower extremity are graded on the Medical Research Council (MRC) scale of 0–5.

*BERA* brainstem electric response audiometry, *B/L* bilateral, *EMG* electromyography, *FDI* first dorsal interosseous, *m* months, *MRI* magnetic resonance imaging, *ND* no data, *OFC* occipital frontal circumference, *TA* tibialis anterior, *wks* weeks, *y* years, *z* *z* score.

The 12-year-old sister of patient 1–1 (patient 1–2) had similar complaints that started at 10 years of age with difficulty holding a cup and fine motor impairments in the fingers, right more than left (Table 1 and Supplementary Table 1). She also denied difficulty in walking, climbing stairs and getting up from squatting position. She had premature graying of hair at the age of 4 months. Her past medical history was also notable for a right inguinal herniorrhaphy at 5 years of age. Physical examination revealed wasting of the thenar and hypothenar eminences, along with the interossei bilaterally. She had mild contractures of both thumbs and the left index finger. She could not oppose her thumb with the 4th and 5th fingers. She displayed mild weakness of handgrip. NCS demonstrated low amplitude left median CMAPs and absent right median CMAPs. Needle EMG demonstrated chronic denervation, suggesting the presence of a motor neuropathy or neuronopathy (Supplementary Table 3). A left peroneus muscle biopsy demonstrated neurogenic features.

The younger brother of patients 1–1 and 1–2 (patient 1–3) was asymptomatic. Clinical evaluation at 6 years 6 months showed mild bilateral handgrip weakness (Table 1 and Supplementary Table 1). NCS showed demyelinating and axonal features in the upper extremities (Supplementary Table 4).

Patient 2–1 is the third child born full term to healthy, non-consanguineous parents (Table 1 and Supplementary Table 1). She was a bit slower to walk and toilet train compared to her two older siblings. She had difficulty keeping up with peers in physical education. She was evaluated in the neurology clinic at 8 years 5 months for abnormal gait. She also had impaired balance, difficulty climbing stairs, frequent falls, difficulty squatting, and generalized fatigue. Initial physical exam was notable for prominent distal upper and lower extremity weakness as well as absent ankle reflexes. Muscle bulk was diffusely reduced, with significant atrophy of the thenar eminences and interosseous muscles bilaterally.

She was found to have mild dilated cardiomyopathy. Within a few months, she developed heart failure and required heart transplantation. NCS revealed borderline low amplitude tibial CMAPs for age and essentially normal sensory nerve action potentials (Supplementary Table 5). Needle EMG demonstrated mildly neurogenic motor unit morphology. A gastrocnemius muscle biopsy revealed a neurogenic pattern with fiber type grouping and no inflammation.

Patient 3–1 presented at 11 years with distal upper limb weakness (right > left). She experienced difficulty writing, buttoning, and opening bottle caps. She had generalized tremors. On physical examination, she had significant wasting of both hands (thenar > hypothenar), accompanied by weakness most prominently at the dorsal interossei, and to a lesser extent at the palmar interossei and thenar muscles, with relative sparing of the lumbrical muscles. There were tremors but no fasciculations in the upper extremities. There were mild cerebellar signs, including mild gaze-evoked nystagmus, dysdiadochokinesia, and difficulty tandem walking. NCS and needle EMG demonstrated mixed axonal and demyelinating motor findings corresponding to the areas of weakness on physical examination (Table 1 and Supplementary Table 1).

By 13 years, she had progressive worsening of the upper extremity symptoms, along with new proximal lower extremity weakness of 4 months’ duration, exhibited by inability to stand from a squatting position. She had developed nearly symmetrical weakness of hip extension, thigh adduction, knee flexion, and ankle eversion. There were brisk upper extremity reflexes, diminished patellar reflexes, and absent ankle reflexes bilaterally, with extensor plantar responses. She had mild ataxia and a spastic-ataxic gait, with a normal sensory examination. NCS again showed a mixed demyelinating and axonal pattern of abnormalities in the motor nerves, worse since the prior study (Supplementary Table 6). Needle EMG showed discrete recruitment patterns from all muscles tested (Table 1). Autonomic studies showed abnormal expiration/inspiration and 30:15 ratios, suggestive of parasympathetic dysfunction.

### Genetic and biochemical findings

Singleton WES was performed on patient 1–2. We filtered variants assuming a recessive inheritance pattern and detected the homozygous variant c.1285 A > G in *SLC5A6* predicting the amino acid substitution p.(Ser429Gly) (Tables 1 and 2). The variant is absent from public databases including gnomAD and is predicted to affect function by the in silico tools CADD, REVEL, and M-CAP. Serine 429 is intolerant of variation as predicted by MetaDome (Table 2) [12]. Sanger sequencing confirmed the homozygous *SLC5A6* variant in patients 1–1, 1–2, and 1–3, and also confirmed that each asymptomatic parent harbored the heterozygous variant (Supplementary Fig. 1a).

**Table 2:** Minor allele frequency (MAF) and in silico pathogenicity predictions for *SLC5A6* missense variants.

Nucleotide change (NM_021095.4)	Amino acid change (NP_066918.4)	Genomic position (GRCh37 / hg19)	MAF worldwide (gnomAD v.2.1.1)	Pathogenicity predictions			Genetic tolerance
				CADD	REVEL	M-CAP	
c.368 G > T	p.(Arg123Leu)	2:27430151	0.000004	26	0.778	0.250	Slightly Intolerant
c.485 A > G	p.(Tyr162Cys)	2:27429377	0.00002	29	0.910	0.294	Intolerant
c.1199 G > C	p.(Arg400Thr)	2:27426109	0.000008	22	0.611	0.249	Slightly Intolerant
c.1285 A > G	p.(Ser429Gly)	2:27424933	absent	24	0.737	0.171	Intolerant

The functional impact of the identified variants was predicted by the Combined Annotation Dependent Depletion (CADD) tool, the Rare Exome Variant Ensemble Learner (REVEL) scoring system, and the Mendelian Clinically Applicable Pathogenicity (M-CAP) Score. CADD is a framework that integrates multiple annotations in one metric by contrasting variants that survived natural selection with simulated mutations. Reported CADD scores are phred-like rank scores based on the rank of that variant’s score among all possible single nucleotide variants of hg19, with 10 corresponding to the top 10%, 20 at the top 1%, and 30 at the top 0.1%. The larger the score the more likely the variant has deleterious effects; the score range observed here is strongly supportive of pathogenicity, with all observed variants ranking above ~99% of all variants in a typical genome and scoring similarly to variants reported in ClinVar as pathogenic (~85% of which score >15). REVEL is an ensemble method predicting the pathogenicity of missense variants with a strength for distinguishing pathogenic from rare neutral variants with a score ranging from 0 to 1. The higher the score the more likely the variant is pathogenic. M-CAP is a classifier for rare missense variants in the human genome, which combines previous pathogenicity scores (including SIFT, Polyphen-2, and CADD), amino acid conservation features and computed scores trained on mutations linked to Mendelian diseases. The recommended pathogenicity threshold is >0.025. Genetic tolerance at the affected amino acid position in the protein was predicted by MetaDome [12].

On trio-WES, patient 2–1 was found to have a maternally inherited c.280 C > T, p.(Arg94*) variant and a paternally inherited c.485 A > G, p.(Tyr162Cys) variant in *SLC5A6* (Tables 1, 2). The c.280 C > T, p.(Arg94*) variant has a worldwide minor allele frequency (MAF) of 0.00002 (gnomAD) and has been described to affect function [9]. The unreported variant c.485 A > G has a worldwide MAF of 0.00002 (gnomAD) and is predicted to affect function. Tyrosine 162 is predicted to be intolerant of variation (Table 2). The variants in the trio were confirmed via Sanger sequencing (Supplementary Fig. 1b).

Singleton WES in patient 3–1 revealed the same homozygous *SLC5A6* variant as identified in family 1: c.1285 A > G, p.(Ser429Gly) (Tables 1, 2). Sanger sequencing confirmed the homozygous variant in patient 3–1 and the heterozygous variant in the unaffected parents (Supplementary Fig. 1c).

Analysis of exome sequencing data in patients 1–2 and 3–1 for regions of homozygosity (ROHs) revealed 15 ROHs with a total of 54 Mb in patient 1–2 (Supplementary Table 7) and 14 ROHs with a total of 67.07 Mb in patient 3–1 (Supplementary Table 8) suggesting parental consanguinity in both families. We identified a homozygous region on chromosome 2 of 7.34 Mb (chr2:25,376,564-32,713,706) and of 5.9 Mb (chr2:22,865,260-28,762,031) in patient 1–2 and 3–1, respectively, that includes the *SLC5A6* variant c.1285 A > G (Supplementary Tables 7, 8). The region contains a 3.39-Mb (chr2:25,376,564-28,762,031) shared haplotype in both patients suggesting remote relatedness (Supplementary Table 9).

GeneMatcher [13] and medical care of the patients 1–1, 1–2, 1–3, and 3–1 by one of the authors (S.N.) permitted co-ordinated investigation of the families. Metrics from gnomAD show fewer than expected missense variants (o/e = 0.76), indicating that *SLC5A6* is slightly intolerant of missense variants [14]. We believe the biallelic *SLC5A6* variants to underlie the motor neuropathies in the five patients described here as the detected variants were absent or rare in population databases and predicted to affect function, and biallelic *SLC5A6* variants have been reported to cause disease [9–11].

Biotin deficiency was confirmed in serum of patient 1–2 (Table 3). As this biochemical test was not available for the patients from India, we determined that pyruvate carboxylase activity was similar between patient 1–2 and control fibroblasts (Supplementary Fig. 2). Plasma biotinidase activity in all patients was normal (Table 3). We did not find any rare variants affecting function in *BTD* in the exome data of patients 1–2, 2–1, and 3–1. The exclusion of biotinidase deficiency in all patients support the association of biallelic *SLC5A6* variants with motor neuropathies in the current cohort.

**Table 3:** Interval clinical improvement in five patients with biallelic *SLC5A6* variants.

	Patient 1–1	Patient 1–2	Patient 1–3	Patient 2–1	Patient 3–1
Age at initiation of therapy	15 y 1 m	12 y 10 m	7 y 10 m	9 y 3 m	13 y 6 m
Serum vitamin levels before treatment	Not available	Not available	Not available	Vitamin B7 (biotin) <100.0 pg/mL [100.0–2460.2] Vitamin B5 (pantothenic acid) 44.79 µg/L [3.45–229.2 for children 1–10 y]	Not available
Initial therapy	Biotin 5 mg BID	Biotin 5 mg BID	Biotin 5 mg BID	Biotin 5 mg BID Pantothenic acid 250 mg QD Lipoic acid 150 mg QD	Riboflavin 50 mg BID Pyridoxine OD Co Q 30 mg BID Carnitine 330 mg BID Vitamin E 400 mg BID
Interval Evaluation	No significant clinical changes	No significant clinical changes	No significant clinical changes	Her stamina and strength improved. She is able to walk without tripping	No significant clinical changes
Therapy adjustments	Biotin 100 mg daily Pantothenic acid 500 mg daily Lipoic acid 300 mg daily	Biotin 100 mg daily Pantothenic acid 500 mg daily Lipoic acid 300 mg daily	Biotin 100 mg daily Pantothenic acid 500 mg daily Lipoic acid 300 mg daily	Biotin 10 mg BID Pantothenic acid 250 mg BID Lipoic acid 150 mg BID	Biotin 10 mg TID Lipoic acid 100 mg BID (in addition to above mentioned vitamins)
Most recent clinical evaluation^a^					
Interval history	Hand grip and finger strength improved	Hand grip and finger strength improved	No significant clinical changes	She continued to improve steadily; her stamina and strength improved significantly; she is now able to keep up with her peers	Patient reported improvement in strength and coordination, however, her physical exam was unchanged
Age/length/weight (z score)	15 y 8 m 149 cm (−3.16) 36 kg (−2.07)	13 y 6 m 141 cm (−3.63) 28 kg (−2.68)	8 y 6 m 125 cm (−0.98) 20 kg (−2.32)	10 y 141.2 cm (0.45) 27.3 kg (−1.03)	13 y 9 m 50 kg (0.04)
Vitamin levels	Biotinidase 4.61 [4.5–12.0 nmol/mL/min]	Biotinidase 5.19 [4.5–12.0 nmol/mL/min]	Biotinidase 4.91 [4.5–12.0 nmol/mL/min]	Biotinidase 6.7 [5.1–11.9 nmol/mL/min] Vitamin B5 (pantothenic acid): 477 [12.9–253.1 ng/mL]	Biotinidase 5.58 [4.5–12.0 nmol/mL/min]
Neuromuscular exam findings^a^					
Muscle bulk	Muscle bulk reduced throughout; atrophy of thenar and hypothenar eminence (more prominent on the right hand); bilateral atrophy of interosseous muscles	Atrophy of thenar and hypothenar eminence (more prominent on the right hand); bilateral atrophy of interosseous muscles	Bilateral atrophy of interosseous muscles (more prominent on the right hand)	Muscle bulk reduced throughout; bilateral atrophy of thenar eminence and interosseous muscle	Atrophy of thenar and hypothenar eminence (more prominent on the right hand); thenar > hypothenar atrophy; interosseous muscles atrophy noted
Neck	Flexion & extension 5/5	Flexion & extension 5/5	Flexion & extension 5/5	Flexion & extension 5/5	Flexion & extension 5/5
Upper extremity	Deltoids 5 Triceps 5 Biceps 5 Wrist flexion & extension 5/5 Extensor pollicis brevis 4 Extensor pollicis longus 4 Palmar interosseus 4 Abductor digitiminimi 4 Abductor pollicis brevis 4	Deltoids 5 Triceps 5 Biceps 5 Wrist flexion & extension 5/5 Extensor pollicis brevis 4 Extensor pollicis longus 4 Palmar interosseus 4 Abductor digitiminimi 4 Abductor pollicis brevis 4	Deltoids 5 Triceps 5 Biceps 5 Wrist flexion & extension 3/3 Extensor pollicis brevis 2 Extensor pollicis longus 2 Palmar interosseus 3 Abductor digitiminimi 2 Abductor pollicis brevis 3	Deltoids 5 Triceps 5 Biceps 4 Wrist flexion & extension 5/5 Extensor digitorum communis 4 First dorsal interosseous 5 Abductor digitiminimi 5 Abductor pollicis brevis 4 Grip 4	Deltoids 5 Triceps 5 Biceps 5 Wrist flexion & extension 5/5 Extensor digitorum communis 4 Palmar and dorsal interosseous 3 abductor pollicis 4 adductor pollicis 4 opponens pollicis 4 flexor pollicis brevis Lumbrical 5 Grip 4+
Lower extremity	Gluteus medius 4 Gluteus maximus 4 Iliopsoas 4 Quadriceps 5 Hamstrings 5 Tibialis anterior 5 Tibialis posterior 5 Gastrocnemius 5	Gluteus medius 4 Gluteus maximus 4 Iliopsoas 4 Quadriceps 5 Hamstrings 5 Tibialis anterior 5 Tibialis posterior 5 Gastrocnemius 5	Gluteus medius 5 Gluteus maximus 5 Iliopsoas 5 Quadriceps 5 Hamstrings 5 Tibialis anterior 5 Tibialis posterior 5 Gastrocnemius 5	Gluteus medius & maximus 5 Iliopsoas 5 Quadriceps 5 Hamstrings 5 Tibialis anterior 4+ Tibialis posterior 4 Peroneus longus/brevis 5 Extensor hallucis longus 5 Gastrocnemius 5	Gluteus medius & maximus 5 Iliopsoas 4 Quadriceps 5 Hamstrings 4− Tibialis anterior 4 Tibialis posterior 4 Peroneus longus/brevis 4 Extensor hallucis longus 4 Gastrocnemius 5
Reflexes	Reflexes reduced throughout	Reflexes reduced throughout	Normal reflexes	2+ in upper extremity and lower extremity except trace at ankles; down going plantar response bilaterally	2+ in upper extremity Reduced in lower extremity and absent at ankles bilaterally
Gait	Normal	Normal	Normal	Mild abnormal gait: normal toe walking and slightly difficult heel and tandem walking	Ataxic gait with minimal waddling; unable to perform tandem walking

*BID* twice a day, *cm* centimetre, *kg* kilogram, *L* liter, *m* months, *mg* milligram, *µg* microgram, *min* minute, *ml* milliliter, *nmol* nanomoles, *pg* picogram, *OD* once a day, *TID* three times a day, *y* years.

^a^Most recent clinical evaluation was performed at 1 month after last therapy adjustments for patient 1–1, patient 1–2, and patient 1–3, 9 months of therapy initiation for patient 2–1, and 3 months after last therapy adjustments for patient 3–1. Due to restrictions related to the SARS-CoV-2 pandemic, these medications could not be obtained by nor administered to patients 1–1, 1–2, and 1–3 for six months prior to this last dose adjustment.

### Human SMVT 3D homology modeling

**Fig. 1:**
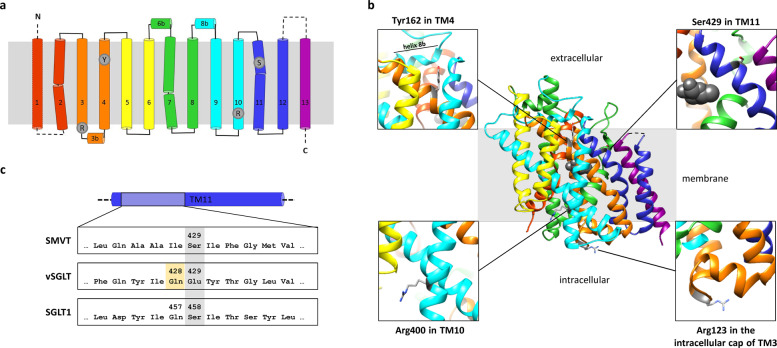
Secondary structure and 3D model of SMVT and location of amino acid residues substituted in affected individuals. **a** Predicted secondary structure of SMVT with 13 membrane spanning α-helices (numbered cylinders in rainbow color). Extra- and intracellular loops connecting the transmembrane α-helices (TMs) are visualized by lines. Dashed lines were used for helices and loops which could not be modeled (**b**). Amino acid residues affected by variants in patients are shown as gray dots (R in the intracellular cap of TM3: Arg123; Y in TM4: Tyr162; R in TM10: Arg400; S in TM11: Ser429). **b** 3D model of SMVT and its orientation in the membrane, generated with SWISS-MODEL based on the crystal structure of vSGLT (PDB ID: 3DH4) [15, 16]. The model includes residues 65 to 550 and omits TM1 and the large extracellular loop between TM12 and TM13 (dashed lines in **a**). The helices are rainbow-colored. The possible substrate localization is visualized by gray spheres. Arg123, Tyr162, Arg400, and Ser429 are colored in gray and represented as stick models. The insets show magnifications to highlight the location of each of the changed residues. The helix 8b is indicated in the inset in the upper left corner. Model modifications and creation of the image were done using UCSF chimera. **c** Partial amino acid sequence alignment of human SMVT (SLC5A6), vSGLT and human SGLT1 (SLC5A1). Full multiple sequence alignment is shown as Supplementary Fig. 4. TM11 is shown as a cylinder above the alignment. Ser429 and the corresponding residues in vSGLT and SGLT1 are highlighted in gray. Gln428, a residue involved in substrate binding in vSGLT [15, 26], is highlighted in yellow.

Knowledge of the human SMVT structure-function relationship is limited as structural approaches using purified SMVT are missing [2]. SMVT together with the sodium:galactose symporter SGLT1, encoded by *SLC5A1*, and the sodium:iodide symporter NIS, encoded by *SLC5A5*, belong to the SSS family that contain 10–14 transmembrane α-helices (TM) [1]. To gain insight into the effects of SLC5A6 amino acid substitutions reported previously and here (Table 2), we aligned the amino acid sequence of human SMVT with the sodium:galactose symporter from *Vibrio parahaemolyticus* (vSGLT) (Supplementary Fig. 3). This alignment confirmed 13 TMs for SMVT with an extracellular N-terminus, a large extracellular loop between TM12 and TM13, and a cytoplasmic C-terminus (Fig. 1a and Supplementary Fig. 3) [2].

We next generated a 3D model of SMVT using vSGLT as template (Fig. 1b) [15, 16]. The structure of the N- and C-terminus, TM1, the TM1-TM2 loop, and the large TM12-TM13 loop could not be predicted due to missing residues in the vSGLT crystal structure and limitations of homology predictions for random coiled regions (shown as dashed lines in Fig. 1a, b). The previously reported disease-associated substitutions p.(Arg123Leu) and p.(Arg400Thr) (Table 2) and p.(Tyr162Cys) and p.(Ser429Gly) identified here do not cluster in a specific SMVT region (Fig. 1a, b). Arg123 is located at the intracellular cap of TM3 and Arg400 in the cytoplasmic facing region of TM10 (Fig. 1a, b). TM10 in vSGLT has a supporting function by stabilizing the central helices [15]. Tyr162 is located at the cytoplasmic facing region of TM4 (Fig. 1a, b). In vSGLT, TM4 is one of the seven central helices. The correct positioning of the central helices extends the cavity from just below the substrate-binding site to the intracellular space and contributes to side chain interactions for ligand sensitivity [15]. Except this, the extracellular facing ends of TM2 and TM4 form extensive contacts with helix 8B, which is localized in the extracellular linker of TM8 and TM9 and straddles the membrane plane [15]. In the SMVT model, Tyr162 is in spatial proximity to helix 8b at the extracellular facing region of TM4 (Fig. 1b). Substitution of a large hydrophobic amino acid like tyrosine to a smaller amino acid like cysteine could cause a loss of side chain interactions [17]. Correct positioning of TM4 in the membrane or the interaction between TM4 and helix 8b could be affected. Ser429 is located in TM11 (Fig. 1a–c) which is probably involved in substrate binding. Importantly, structural rearrangement of TM11 is possibly important for substrate transport [15]. Substitution of a polar serine residue by a small and flexible glycine could affect the arrangement of the transmembrane helices and/or substrate binding.

### Functional studies of the *SLC5A6* variants p.(Tyr162Cys) and p.(Ser429Gly)

**Fig. 2:**
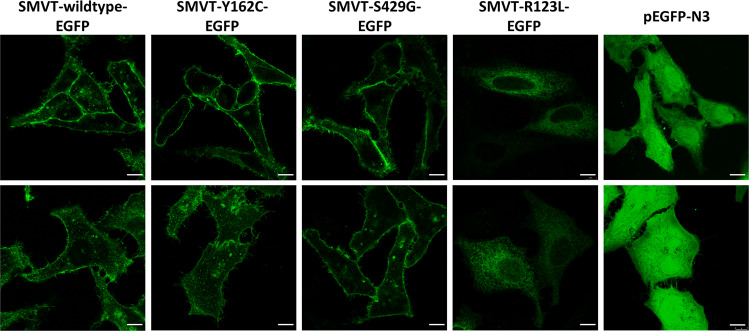
Subcellular localization of ectopically expressed SMVT wildtype and mutants. HeLa cells were transiently transfected with SLC5A6-EGFP expression constructs as indicated or the pEGFP-N3 empty vector, cultivated for 48 h, fixed, and embedded in mounting solution. Images were acquired with a confocal microscope. Representative images of three independent experiments are shown. Scale bar, 10 µm.

To experimentally analyze the effect of the *SLC5A6* variants p.(Tyr162Cys) and p.(Ser429Gly) on SMVT function, we transiently transfected HeLa cells with SLC5A6-EGFP wildtype construct and the mutant constructs SLC5A6-Y162C-EGFP, SLC5A6-S429G-EGFP, and SLC5A6-R123L-EGFP and determined the subcellular distribution of the SMVT by immunofluorescence analysis. We used the SLC5A6-R123L-GFP construct as control as the previously reported p.(Arg123Leu) variant causes intracellular retention of the SMVT [9]. HeLa cells expressing EGFP alone showed an even green fluorescence in the cytoplasm (Fig. 2), while wild-type SLC5A6-EGFP displayed plasma membrane localization (Fig. 2). The two EGFP-tagged SLC5A6 mutants Y162C and S429G were localized at the plasma membrane (Fig. 2), while the SLC5A6-R123L-EGFP mutant was mainly present in the cytoplasm (Fig. 2). These data show that p.(Tyr162Cys) and p.(Ser429Gly) do not affect SMVT plasma membrane localization that is contrast to p.(Arg123Leu) that leads to intracellular retention of the transporter.

### Treatment outcomes

Upon identification of biallelic *SCL5A6* variants, patients 1–1, 1–2, and 1–3 were treated with biotin (5 mg twice daily). After 6 months of treatment, only patient 1–2 had slight improvement in hand strength. Therapy was later extended to biotin 10 mg, pantothenic acid 250 mg, and lipoic acid 150 mg, all dosed twice daily. The most recent regimen was biotin 100 mg, pantothenic acid 500 mg, and lipoic acid 300 mg, all dosed daily. After one month of treatment, hand grip and finger strength were improved in patients 1–1 and 1–2, while patient 1–3 was unchanged (Table 3). Due to restrictions related to SARS-CoV-2, these medications were unavailable to the three siblings for six months prior to this last dose adjustment.

In patient 2–1, biotin (5 mg twice daily), pantothenate (250 mg daily), and α-lipoic acid (150 mg daily) were initiated at 9 years 3 months (Table 3). These doses were doubled 3 months later. At 9 months, the child exhibited marked improvement in weight, height, balance, muscle strength, and exercise tolerance. Her gait was near normal at the last follow-up and she was able to keep up with her peers. However, she had residual mild distal upper extremity weakness.

At the age of 13 years, patient 3–1 was empirically treated with intravenous methylprednisolone for 5 days followed by an oral steroid taper. She began supplementation with riboflavin, coenzyme Q10, pyridoxine, vitamin E, and carnitine (Table 3). There was minimal improvement in gait and coordination over the next month. Upon identification of the *SLC5A6* variant, she was initiated on biotin and α-lipoic acid (Table 3). Five months later, she reported improvement in gait and coordination.

## Discussion

Dysfunction of the Na^+^:vitamin transporter causes phenotypic heterogeneity, including failure to thrive, developmental delay or early normal development followed by developmental regression, seizures, diarrhea or vomiting, immunodeficiency, and/or osteopenia [9–11]. We further expand the phenotypic spectrum associated with biallelic *SLC5A6* variants by reporting five individuals from three families with pleomorphic motor neuropathies that had both axonal and demyelinating features. Findings on NCS were sometimes asymmetric, suggesting that the nerve damage may be non-uniform in some instances. While the presentations in the patients reported here define the mild end of the spectrum, the phenotypes previously reported patients are more severe [9–11]. Biotin deficiency was detected in serum of patient 2–1, while serum vitamin levels could not be determined in the four affected individuals from India (Table 3). Measurement of circulating biotin in serum and plasma is not a reliable measure of status because depressed serum biotin is not consistently observed in biotin deficiency [18]. By measuring the activity of the biotin-dependent pyruvate carboxylase in fibroblasts of patient 1–2 we did not obtain evidence for biotin deficiency in this patient. This was not surprising as normal skin-derived cells show low sensitivity to biotin depletion [19]. In a boy with biotin dependency and suspected inherited defect in biotin transport, normal carboxylase activities in fibroblast extracts were identified. An acute illness elicited a severe metabolic disorder with an encephalopathic episode at 18 months that improved rapidly with biotin supplementation [20]. In retrospect, this patient likely had biallelic *SLC5A6* variants although *SLC5A6* cDNA sequencing did not reveal variants affecting function [20]. A previously reported patient with biallelic *SLC5A6* variants had a severe metabolic crisis with encephalopathy [10]. These data suggest that patients carrying biallelic *SLC5A6* variants with a severe impact on SMVT function can present with a state of precarious biotin homeostasis that can lead to life-threatening metabolic crises.

Mice with intestine-specific *Slc5a6* knockout exhibit growth failure, decreased bone density and length, lethargy, hunched back posture, and gut inflammation [21, 22]. Biotin and pantothenic acid supplementation rescued the phenotype [23]. Two previously reported patients with biallelic *SLC5A6* variants also showed failure to thrive [9, 10] and triple vitamin replacement therapy, likely via a simple diffusion mechanism, had beneficial effects in the three live patients [9–11]. In our cohort, patient 2–1 showed unequivocal improvement with vitamin therapy; the patients in families 1 and 3 did not show consistent improvements, indicating that responses to vitamin therapy are not uniform.

Four of the nine patients with biallelic *SLC5A6* variants reported to date (including the current cohort) carry early nonsense or frameshift variants together with a missense variant [9, 11]. One patient has an early frameshift in combination with a late frameshift variant [10], and four patients reported here are homozygous for the same missense variant. Is there any possible correlation between genotypes and phenotypes? One individual carries the null allele p.(Val141Alafs*34) as it leads to nonsense-mediated mRNA decay (NMD) [11], similar to the nonsense variant p.(Arg94*) reported in another individual [9]. In contrast, the late frameshift variant p.(Gln622Argfs*51) is located in the last *SLC5A6* exon, likely escapes NMD and leads to production of a C-terminally truncated SMVT. The relatively mild phenotype in the patient with p.(Val141Alafs*34) and p.(Gln622Argfs*51) variants suggests residual functioning of the C-terminally altered vitamin transporter [10]. Consistent with this, the C-terminus of SMVT is important for plasma membrane targeting [24, 25]. p.(Arg123Leu) and p.(Arg400Thr) impair biotin uptake by the ectopically expressed SMVT mutants [9, 11] and SMVT-Arg123Leu is retained in the endoplasmic reticulum [9]. The data suggest that the four reported patients with a multisystemic disease and/or near-fatal events carry a null allele in trans with a second allele that produces a functionally impaired SMVT transporter [11].

It is plausible that the p.(Tyr162Cys) and p.(Ser429Gly) variants identified in individuals affected by motor neuropathies have a milder effect on Na^+^:vitamin transporter function. Our studies showed that p.(Tyr162Cys) and p.(Ser429Gly) did not impact plasma membrane targeting of the SMVT, suggesting that both SMVT mutants have reduced function, i.e., a lower catalytic rate. This assumption is corroborated by the finding that Ser429 in SMVT corresponds to Glu429 in vSGLT (Fig. 1c), a residue adjacent to Gln428 (Fig. 1c), which is a key residue involved in substrate binding [15, 26]. An amino acid substitution changing the corresponding glutamine, Gln457, to arginine in human SGLT1 (Fig. 1c) and affecting function has been reported. SGLT1-Gln457Arg is present at the plasma membrane and binds the substrate but does not transport the substrate through the membrane [27, 28]. The effect of the p.(Tyr162Cys) change on SMVT function is difficult to predict. There are no disease-associated amino acid changes in SGLT1 or NIS known to date that affect the corresponding residue. As Tyr162 is in spatial proximity to helix 8b at the extracellular face of TM4 (Fig. 1b), we searched for amino acid substitutions affecting function located in the extracellular helix 8b of other sodium:solute symporters. In SGLT1 substitutions changing Tyr366 and Leu369 and affecting function are located in the extracellular helix 8b, which is in spatial proximity to the extracellular facing end of TM4 [29, 30]. Functional studies revealed a reduced uptake of the substrate by the SGLT1-Leu369Ser mutant [29]. A similar effect could be possible for the SMVT-Y162C mutant. Variants affecting function in *SLC5A5* that only impair transport activity of the encoded NIS have been reported [31]. Similarly, missense variants affecting function in *SLC25A46*, encoding a mitochondrial carrier, can destabilize the carrier, while other missense variants affect the formation/stability of higher molecular weight complexes [32]. Together, missense variants in transporters can cause loss of function, while other amino acid changes leave the protein intact and impair its function.

Is there any link between reduced levels of biotin, pantothenic acid and/or α-lipoic acid and neuropathy? One previously reported patient with biallelic *SLC5A6* variants developed mixed axonal and demyelinating sensorimotor peripheral neuropathy from 3 years of age on that resolved post vitamin treatment. The patient’s sibling died in early childhood. Histopathological analysis demonstrated peripheral nerves with axonal irregularity and patchy denervation atrophy in skeletal muscle biopsy [11]. These findings suggest that reduced biotin, pantothenic acid and/or α-lipoic acid levels may be neurotoxic and contribute to the peripheral neuropathies seen in some individuals with biallelic *SLC5A6* variants. Various combinations of peripheral neuropathy, myelopathy, spastic paraparesis, and optic neuropathy have been described in individuals with biallelic *BTD* variants. Partial reversal of symptoms in these patients with biotin therapy underscores a role of biotin deficiency in peripheral neuropathy [33]. Impaired intestinal biotin uptake has been hypothesized to contribute to peripheral neuropathy in individuals with alcoholism [6, 34]. Successful biotin and/or α-lipoic acid supplementation in individuals with peripheral neuropathy associated with diabetes and alcoholism further supports the association of peripheral neuropathy with reduced levels of these nutrients [35–37].

In conclusion, we report biallelic *SLC5A6* variants affecting function in individuals with motor neuropathies. Plasma membrane localization of the SMVT-Y162C and SMVT-S429G mutants underscores reduced but not abolished function of the multivitamin transporter. Prompt molecular genetic diagnosis is of importance for these patients as vitamin supplementation has a therapeutic benefit.

## Supplementary information

Supplemental Material

## Data Availability

All data generated or analysed during this study are included in this published article and its Supplementary Information files.
