# Supplementary material for: Novel biallelic variants expand the SLC5A6-related phenotypic spectrum
Source: Eur J Hum Genet. 2022 Jan 11;30(4):439–49. doi: 10.1038/s41431-021-01033-2 (PMC8747999; doi:10.1038/s41431-021-01033-2)
Supplement: Supplementary file 1 — Supplemental Material [file 41431_2021_1033_MOESM1_ESM.pdf]

Supplementary Information

Methods

Whole-exome sequencing and variant filtering

Genetic studies in patients 1-1, 1-2, and 1-3 (siblings of family 1) and healthy parents were performed clinically. Whole-exome sequencing (WES) was performed on genomic DNA extracted from leukocytes of patient 1-2 (Centogene): double stranded DNA capture baits against approximately 36.5 Mb of the human coding exome (targeting >98% of the coding RefSeq and Gencode v28 regions, which was obtained from the human genome build GRCh37/hg19 on May 2018) were used to enrich target regions from fragmented genomic DNA with the Twist Human Core Exome Plus kit. The generated library was sequenced on an Illumina platform to obtain at least 20x coverage depth for >98% of the targeted bases. An in-house bioinformatics pipeline was used for variant detection. Sequence reads were aligned to the human reference assembly (GRCh37/hg19) using the Burrows Wheeler Aligner (BWA mem, v0.7.17-r1188) (1). Genetic variants were detected with the Genome Analysis Toolkit (GATK, v3.8) (2) and annotated using ANNOVAR (v2018-04-16) (3). Private (absent in public database including gnomAD) and rare (with minor allele frequency <0.1% and not present in the homozygous state in public databases) exonic and intronic variants at exon-intron boundaries ranging from -10 to +10 were retained. Remaining variants were then prioritized by pathogenicity assessment using the *in silico* tools CADD, REVEL, and M-CAP (4-6).

Trio-WES was performed at a clinical genetic test facility in the United States for patient 2-1 and parents (family 2). Using genomic DNA from the proband and parents, the exonic regions and flanking splice junctions of the genome were captured using the IDT xGen Exome Research Panel v1.0. Massively parallel (NextGen) sequencing was performed using an Illumina system with 100bp or greater paired-end reads. Reads were aligned to human genome build GRCh37/hg19 and analyzed for sequence variants using a custom-developed analysis tool. Additional sequencing technology and variant interpretation protocol has been previously described (7). The general assertion criteria for variant classification are publicly available on the GeneDx ClinVar submission page.

Genomic DNA of patient 3-1 and her parents was extracted from whole blood samples by commercially available kit [DNeasy Blood &amp Kit (QIAGEN)]. Singleton WES was performed in patient 3-1. The coding genomic regions were captured using Twist Human Core Exome Plus kit followed by massively parallel sequencing using Illumina sequencing platforms. Target regions had an average coverage of ~120X. The quality of the data was assured by FastQC check (https://www.bioinformatics.babraham.ac.uk/projects/fastqc/). With the use of an in-house build bioinformatics pipeline [integrating BWA-MEM (v0.7.15)] (1) and Genome Analysis Toolkit (GATK v3.6) (8), reads were aligned to the human genome build GRCh37/hg19 and variant calling and annotation (ANNOVAR) (3) was performed by integrating an in-house pipeline (9). We filtered for rare variants (allele frequency <1%) based on gnomAD database (10) and local population datasets (9). Remaining exonic and splicing variants were considered for further analysis and variant prioritization.

Sanger sequencing permitted *SLC5A6* variant validation and segregation analysis in all three families. *SLC5A6* variants were described according to the GenBank reference sequences NM_021095.4 and NP_066918.4. The *SLC5A6* variants were submitted to the LOVD database (https://databases.lovd.nl/shared/genes/SLC5A6), with LOVD Variant IDs 0000784069, 0000784070, 0000784071, 0000784072, and 0000813612.

Regions of homozygosity

Homozygous regions in patients were identified from exome sequencing data with the use of the AutoMap (v1.0) tool (11). The shared homozygous regions between the two patients 1-2 and 3-1 were calculated by using in build option (‘--common) by AutoMap.

Pyruvate carboxylase activity assay

Fibroblasts were cultured in Dulbecco’s modified Eagle medium (DMEM; Thermo Fisher Scientific; without biotin) supplemented with 10% fetal bovine serum (FBS; GE Healthcare; unknown biotin

concentration) and penicillin-streptomycin (100 U/ml and 100 µg/ml, respectively; Thermo Fisher Scientific; without biotin). 500,000 fibroblasts were homogenized, 2.5-fold diluted, and 5 µL sample was used to determine pyruvate carboxylase (PC) activity using the PC activity colorimetric assay kit (BioVision) according to the manufacturer's protocol. Initial sample protein concentration was determined using the Pierce BCA Protein Assay Kit (Thermo Fischer Scientific) according to the manufacturer's protocol. Unit definition: One unit of pyruvate carboxylase activity is the amount of enzyme that consumes 1.0 µmol NADH per min at pH 7.4 at 37°C. Statistical analysis was performed via one-way analysis of variance (ANOVA) followed by a Dunnett's post hoc test for multiple comparisons. A p value of less than 0.05 was considered statistically significant.

Protein structure homology modeling

Multiple sequence alignments of human SMVT (UniProtKB/Swiss-Prot accession number: Q9Y289), human SGLT1 (UniProtKB/Swiss-Prot accession number: P13866), and human NIS (UniProtKB/Swiss-Prot accession number: Q92911) with SGLT from *Vibrio parahaemolyticus* (UniProtKB/Swiss-Prot accession number: P96169) were generated using the Clustal Omega online server.

The FASTA sequence of human SMVT (Q9Y289) was uploaded to the SWISS-MODEL server https://swissmodel.expasy.org/ and was aligned to the template vSGLT (PDB: 3DH4) with an identity of 22.54% (ClustalW). Modification of the generated 3D model and creation of the images were done using UCSF chimera. The model includes residues from 65 to 550 and misses TM1 and the large extracellular loop between TM12 and TM13 due to missing residues in the vSGLT crystal structure and limitations of homology predictions for random coiled regions. The secondary structure model of SMVT is based on the sequence alignment of human SMVT (Q9Y289) with the amino acid sequence of vSGLT used for the crystal structure 3DH4 and with that of vSGLT used for the crystal structure 2XQ2.

Supplementary figures

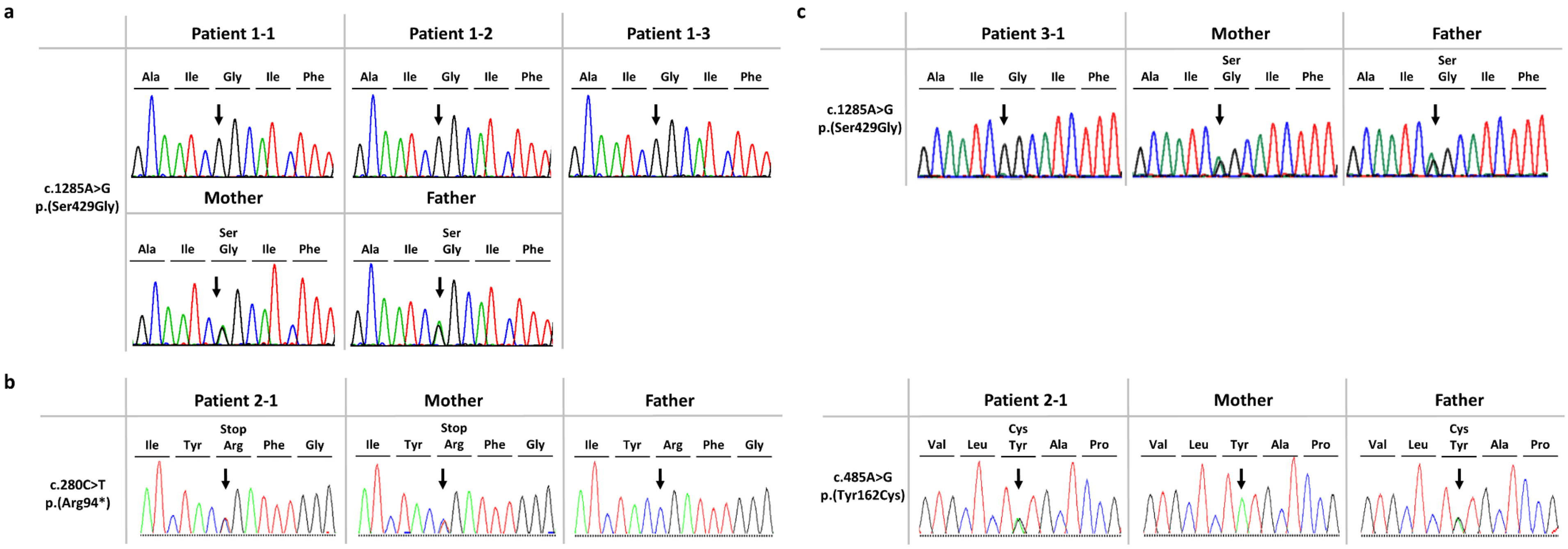

Supplementary Fig. 1 *SLC5A6* variant validation in families 1, 2, and 3

a Partial sequence electropherograms showing the presence of the *SLC5A6* variant c.1285A>G/p.(Ser429Gly) in leukocyte-derived DNA of patients 1-1, 1-2, 1-3, and their parents. Patients 1-1, 1-2, 1-3 are homozygous and parents are heterozygous carriers.

b Partial sequence electropherograms showing the compound heterozygous *SLC5A6* variants c.280C>T/p.(Arg94*) (left) and c.485A>G/p.(Tyr162Cys) (right) in patient 2-1. The mother carries the heterozygous c.280C>T/p.(Arg94*) variant and the father the heterozygous c.485A>G/p.(Tyr162Cys) variant.

c Partial sequence electropherograms showing the *SLC5A6* variant c.1285A>G/p.(Ser429Gly) in leukocyte-derived DNA of patient 3-1 and her parents. Patient 3-1 is homozygous and mother and father are heterozygous.

Arrows point to the mutated nucleotides. Codons and encoded amino acids (three-letter code) are indicated above the sequences.

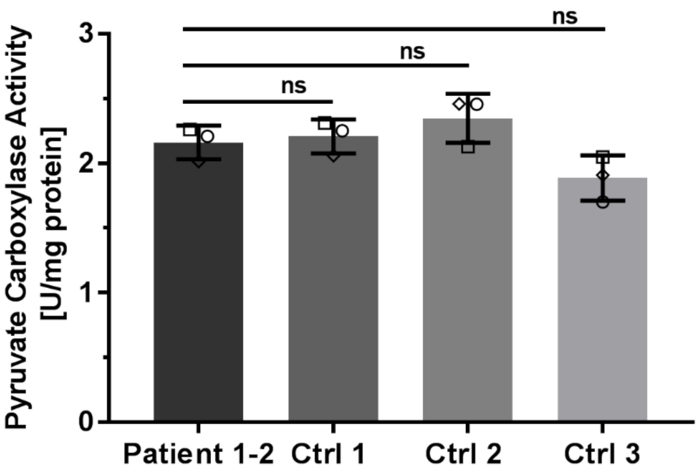

Supplementary Fig. 2 Pyruvate carboxylase activity in patient 1-2 and control fibroblasts

Pyruvate carboxylase activity in patient 1-2 and control fibroblasts in U/mg protein. The mean ± SD and single data points of three independent experiments are shown. Ctrl: control; ns: not significant; U: units.

a

```
                         TM2                         TM3
SMVT    MADRKMGCLPVALSLLATFQSAVAILGVPSEIYRFGTQYWFLGCCYFLGLLIPAHIFIPV 120
3DH4    XAGKSLPWWAVGASLIAANISAEQFIGMSGSGYSIGLAIASYEWMSAITLIIVGKYFLPI 61

                 3b                  TM4
SMVT    FYRLHLTSAYEYLELRFNKTVRVCGTVTFI-FQMVIYMGVVLYAPSLALNAVTGFDLWLS 179
3DH4    FIEKGIYTIPEFVEKRFNKKLKTILAVFWISLYIFVNLTSVLYLGGLALETILGIPLMYS 136

          TM5                        TM6                       6b
SMVT    VLALGIVCTVYTALGGLKAVIWTDVFQTLVMFLGQLAVIIVGSAKVGGLGRVWAVASQH- 238
3DH4    ILGLALFALVYSIYGGLSAVVWTDVIQVFFLVLGGFMTTYMAVSFIGGTDGWFAGVSKMV 196

                                                    TM7
SMVT    ----GRISGFELDPDPF-VRHTFWTLAFG-GVFMMLSLYGVNQAQVQRYLSSRTEKAAVL 293
3DH4    DAAPGHFEMILDQSNPQYMNLPGIAVLIGGLWVANLYYWGFNQYIIQRTLAAKSVSEAQK 373

               TM8                                       8b
SMVT    SCYAVFPFQQVSLCVGCLIGLVMFAYYQEYPMSI---------QQAQAAPDQFVLYFVMD 343
3DH4    GIVFAAFLKLIVPFLVVLPGIAAYVITSDPQLMASLGDIAATNLPSAANADKAYPWL-TQ 256

                              TM9
SMVT    LLKGLPGLPGLFIACLFSGSLSTISSAFNSLATVTMEDLIRPWF-PEFSEARAIMLSRGL 402
3DH4    FL--PVGVKGVVFAALAAAIVSSLASMLNSTATIFTMDIYKEYISPDSGDHKLVNVGRTA 373

          TM10                        TM11                    TM12
SMVT    AFGYGLLCLGMAYISSQMGPVLQAAISIFGMVGGPLLGLFCLGMFFPCANPPGAVVGLLA 462
3DH4    AVVALIIACLIAPMLGGIGQAFQYIQEYTGLVSPGILAVFLLGLFWKKTTSKGAIIGVVA 433

SMVT    GLVMAFWIGIGSIVTSMGSSMPPSPSNGSSFSLPTNLTVATVTTLMPLTTFSKPTGLQRF 522
3DH4    SIPFALFLKFM------------------------------------------------P 445

                   TM13
SMVT    YSLSYLWYSAHNSTTVIVVGLIVSLLTGRMRGRSLNPATIYPVLPKLLSLLPLSCQKRLH 582
3DH4    LSMPFMDQMLYTLLFTMVVIAFTSLSTSINDDD---------------------------

SMVT    CRSYGQDHLDTGLFPEKPRNGVLGDSRDKEAMALDGTAYQGSSSTCILQETSL
3DH4    -----------------------------------------------------
```

b

```
                                                   TM1
SMVT    MSVGVSTSAPLSPTSGTSVGMSTFSIMDYVVFVLLLVLSLAIGLYHACRGW-GRHTVGEL 59
2XQ2    ------------------NIEHGLSFIDIMVFAIYVAIIIGVGLWVSRDKKGTQKSTEDY 44

                                   TM2                     TM3
SMVT    LMADRKMGCLPVALSLLATFQSAVAILGVPSEIYRFGTQYWFLGCCYFLGLLIPAHIFIP 119
2XQ2    FLAGKSLPWWAVGASLIAANISAEQFIGMSGSGYSIGLAIASYEWMSAITLIIVGKYFLP 104

                     3b                  TM4
SMVT    VFYRLHLTSAYEYLELRFNKTVRVCGTVTFI-FQMVIYMGVVLYAPSLALNAVTGFDLWL 178
2XQ2    IFIEKGIYTIPEFVEKRFNKKLKTILAVFWISLYIFVNLTSVLYLGGLALETILGIPLMY 164

           TM5                        TM6                    6b
SMVT    SVLALGIVCTVYTALGGLKAVIWTDVFQTLVMFLGQLAVIIVGSAKVGGLGRVWAVASQH 238
2XQ2    SILGLALFALVYSIYGGLSAVVWTDVIQVFFLVLGGFMTTYMAVSFIGGTDGWFAGVSKM 224

                                                TM7
SMVT    -----GRISGFELDPDPFV-RHTFWTLAFG-GVFMMLSLYGVNQAQVQRYLSSRTEKAAV 291
2XQ2    VDAAPGHFEMILDQSNPQYMNLPGIAVLIGGLWVANLYYWGFNQYIIQRTLAAKSVSEAQ 284

                  TM8                                          8b
SMVT    LSCYAVFPFQQVSLCVGCLIGLVMFAYYQEYPMSI---------QQAQAAPDQFVLYFVM 342
2XQ2    KGIVFAAFLALIVPFLVVLPGIAAYVITSDPQLMASLGDIAATNLPSAANADKAYPWL-T 343

                               TM9
SMVT    DLLKGLPGLPGLFIACLFSGSLSTISSAFNSLATVTMEDLIRPWF-PEFSEARAIMLSRG 401
2XQ2    QFL--PVGVKGVVFAALAAAIVSSLASMLNSTATIFTMDIYKEYISPDSGDHKLVNVGRT 401

          TM10                      TM11
SMVT    LAFGYGLLCLGMAYISSQMGPVLQAAISIFGMVGGPLLGLFCLGMFFPCANPPGAVVGLL 461
2XQ2    AAVVALIIAALIAPMLGGIGQCFQYIQEYTGLVSPGILAVFLLGLFWKKTTSKGAIIGVV 461

        TM12
SMVT    AGLVMAFWIGIGSIVTSMGSSMPPSPSNGSSFSLPTNLTVATVTTLMPLTTFSKPTGLQR 521
2XQ2    ASIPFALFLKFMP----------------------------------------------- 474

                       TM13
SMVT    FYSLSYLWYSAHNSTTVIVVGLIVSLLTGRMRGRSLNPATIYPVLPKLLSLLPLSCQKRL 580
2XQ2    -LSMPFMDQMLYTLLFTMVVIAFTSLSTSINDD--------------------------- 506

SMVT    HCRSYGQDHLDTGLFPEKPRNGVLGDSRDKEAMALDGTAYQGSSSTCILQETSL 635
2XQ2    ------------------------------------------------------
```

Supplementary Fig. 3 Sequence alignments of human SMVT (SLC5A6) with vSGLT

a and **b** Sequence alignment of human SMVT (UniProtKB accession number: Q9Y289) with the amino acid sequence of vSGLT used for the crystal structure 3DH4 (**a**) and with that of vSGLT used for the crystal structure 2XQ2 (**b**). Both alignments were automatically generated by SWISS-MODEL with ClustalW and are the basis for the 2D model in Figure 1a. The alignment of SMVT with 3DH4 is the basis for the 3D structure in Figure 1b. Transmembrane helices (TM1 to TM13) are highlighted in rainbow colors corresponding to the colors shown in Figures 1a and b. Helices 3b, 6b, and 8b are indicated. Amino acid residues affected by variants in SMVT (*SLC5A6*) in patients are colored in red (R123, Y162, R400, S429).

```
P96169|SGLT_VIBPH  ------------------MSNIEHGLSFIDIMVFAIYVAIIIGVGLWVSRDKKGTQKSTE 42
Q9Y289|SC5A6_HUMAN MSVGVSTSAPLS--PTSGTSVGMSTFSIMDYVVFVLLLVLSLAIGLYHACRGW-GRHTVG 57
P13866|SC5A1_HUMAN --MDSSTWSPKTTAVTRPVETHELIRNAADISIIVIYFVVVMAVGLWAMFSTN--RGTVG 56
Q92911|SC5A5_HUMAN --------------MEAVETGERPTFGAWDYGVFALMLLVSTGIGLWVGLARG-GQRSAE 45
                                             .  *  ::.: . :  .:**:        : :.
P96169|SGLT_VIBPH  DYFLAGKSLPWWAVGASLIAANISAEQFIGMSGSGYSIGLAIASYEWMSAITLIIVGKYF 102
Q9Y289|SC5A6_HUMAN ELLMADRKMGCLPVALSLLATFQSAVAILGVPSEIYRFGTQYWFLGCCYFLGLLIPAHIF 117
P13866|SC5A1_HUMAN GFFLAGRSMVWWPIGASLFASNIGSGHFVGLAGTGAASGIAIGGFEWNALVLVVVLGWLF 116
Q92911|SC5A5_HUMAN DFFTGGRRLAALPVGLSLSASFMSAVQVLGVPSEAYRYGLKFLWMCLGQLLNSVLTALLF 105
                     : ..: :    :. ** *:  .:  .:*: .     *           :  :: .  *
P96169|SGLT_VIBPH  LPIFIEKGIYTIPEFVEKRFNKKL-KTILAVFWISLYIF---VNLTSVLYLGGLALETIL 158
Q9Y289|SC5A6_HUMAN IPVFYRLHLTSAYEYLELRFNKTVRVC-----GTVTFIFQMVIYMGVVLYAPSLALNAVT 172
P13866|SC5A1_HUMAN VPIYIKAGVVTMPEYLRKRFGGQRIQVYLSLLSLLLYIF---TKISADIFSGAIFINLAL 173
Q92911|SC5A5_HUMAN MPVFYRLGLTSTYEYLEMRFSRAVRLC-----GTLQYIVATMLYTGIVIYAPALILNQVT 160
                   :*:: .  : :  *::. **.               :*.         ::  .: ::
P96169|SGLT_VIBPH  GIPLMYSILGLALFALVYSIYGGLSAVVWTDVIQVFFLVLGGFMTTYMAVSFIGGTDGWF 218
Q9Y289|SC5A6_HUMAN GFDLWLSVLALGIVCTVYTALGGLKAVIWTDVFQTLVMFLGQLAVIIVGSAKVGGLGRVW 232
P13866|SC5A1_HUMAN GLNLYLAIFLLLAITALYTITGGLAAVIYTDTLQTVIMLVGSLILTGFAFHEVGGYDAFM 233
Q92911|SC5A5_HUMAN GLDIWASLLSTGIICTFYTAVGGMKAVVWTDVFQVVVMLSGFWVVLARGVMLVGGPRQVL 220
                   *: :  :::    .  .*:  **: **::**.:*...:. *       .   :**
P96169|SGLT_VIBPH  AGVSKMVDA----APGHFEMILD---------QSNPQYMNLPGIAVLIGGLWVANLYYWG 265
Q9Y289|SC5A6_HUMAN AVA------------------SQHGRISGFELDPDPFVRHTFWT--LAFGGVFMMLSLYG 272
P13866|SC5A1_HUMAN EKYMKAIPTIVSDGNTTFQEKCYTPRADSFHIFRDPLTGDLPW-PGFIFGMSILTLWYWC 292
Q92911|SC5A5_HUMAN TLA------------------QNHSRINLMDFNPDPRSRYTFWT--FVVGGTLVWLSMYG 260
                                                     :*          :  *  .  *  :
P96169|SGLT_VIBPH  FNQYIIQRTLAAKSVSEAQKGIVFAAFLKLIVPFLVVLPGIAAYVITSDPQLMASLGDIA 325
Q9Y289|SC5A6_HUMAN VNQAQVQRYLSSRTEKAAVLSCYAVFPFQQVSLCVGCLIGLVMFAYYQE---------YP 323
P13866|SC5A1_HUMAN TDQVIVQRCLSAKNMSHVKGGCILCGYLKLMPMFIMVMPGMISRILYTEKIACVVPSECE 352
Q92911|SC5A5_HUMAN VNQAQVQRYVACRTEKQAKLALLINQVGLFLIVSSAACCGIVMFVFYTD---------CD 311
                    :*  :** ::.:. . .  .         :        *:       :
P96169|SGLT_VIBPH  ATNLPSAANADKAYP-WLTQFL--PVGVKGVVFAALAAAIVSSLASMLNSTATIFTMDIY 382
Q9Y289|SC5A6_HUMAN MSIQQAQAAPDQFVLYFVMDLLKGLPGLPGLFIACLFSGSLSTISSAFNSLATVTMEDLI 383
P13866|SC5A1_HUMAN KYCGTKVGCTNIAYPTLVVELM--PNGLRGLMLSVMLASLMSSLTSIFNSASTLFTMDIY 410
Q92911|SC5A5_HUMAN PLLLGRISAPDQYMPLLVLDIFEDLPGVPGLFLACAYSGTLSTASTSINAMAAVTVEDLI 371
                          .  :      : :::    *: *:.::   :. :*: :: :*: :::    *:
P96169|SGLT_VIBPH  KEYISPDSGDHKLVNVGRTAAVVALIIACLIA--PMLGGIGQAFQYIQEYTGLVSPGILA 440
Q9Y289|SC5A6_HUMAN RPWFPEFSEARA-IMLSRGLA-FGYGLLCLGMAYISSQM-GPVLQAAISIFGMVGGPLLG 440
P13866|SC5A1_HUMAN AKVRKRASE-KELMIAGRLFILVLIGISIAWVPIVQSAQSGQLFDYIQSITSYLGPPIAA 469
Q92911|SC5A5_HUMAN KPRLRSLAPRKL-VIISKGLS-LIYGSACLTVAALSSLLGGGVLQGSFTVMGVISGPLLG 429
                          :  :  :  .:    .                 *  ::      . :.  :  .
P96169|SGLT_VIBPH  VFLLGLFWKKTTSKGAIIGVVASIPFALFLKF-----------MPLSMPFMD-QMLYTLL 488
Q9Y289|SC5A6_HUMAN LFCLGMFFPCANPPGAVVGLLAGLVMAFWIGIGSIVTSMGSS-MPPSPSNGS-SFSLPTN 498
P13866|SC5A1_HUMAN VFLLAIFWKRVNEPGAFWGLILGLLIGISRMITEFAYGTGSCMEPSNCPTII-CGVHYLY 528
Q92911|SC5A5_HUMAN AFILGMFLPACNTPGVLAGLGAGLALSLWVALGATLYPPSEQTMRVLPSSAARCVALSVN 489
                    *  *.:*    .  *.. *:  .: :.:   :
P96169|SGLT_VIBPH  FTMVVIAFTSLSTSINDDDPKGISVTSSMFVTDRSF--------------------NIAA 528
Q9Y289|SC5A6_HUMAN LT-VATVTTLMP----------LTTFSKPTGLQRFYSLSYLWYSAHNSTTVIVVGLIVSL 547
P13866|SC5A1_HUMAN FAIILFAISFITIV------------------------------------------VISL 546
Q92911|SC5A5_HUMAN AS-GLLDPALLPANDSSRAPSSGMDASRPALADSFYAISYLYYGALGTLTTVLCGALISC 548
                    :      : :                                              ::
P96169|SGLT_VIBPH  YGIMIVLA--VLYTLFW------------------------------------------- 543
Q9Y289|SC5A6_HUMAN LTGRMRGRSLNPATIYPVLPKLLSLL-PLSCQKRLH-CRSYGQDHLD-T-GLFPEKPRNG 603
P13866|SC5A1_HUMAN LTKPIPDV--HLYRLCWSLRNSKEERI---DLDAEEENIQEGPKETIEIETQVPEKK-KG 600
Q92911|SC5A5_HUMAN LTGPTKRSTLAPGLLWWDLARQTASVAPKEEVAILDDNLVKGPEELP-T-GN---KKPPG 603
                                 :
P96169|SGLT_VIBPH  ------------------------------------------------------------
Q9Y289|SC5A6_HUMAN VLGDSRDKE-----------------AMALDGTA--YQGSSSTCILQE-------TSL-- 635
P13866|SC5A1_HUMAN IFRRAYDLFCGLEQHGAPKMTEEEEKAMKMKMTDTSEKPLWRTVLNVNGIILVTVAVFCH 660
Q92911|SC5A5_HUMAN FLPTNEDRL-----------------FFLGQKELEGAGSWTPCVGHDGGRDQQETNL-- 643

P96169|SGLT_VIBPH  ----
Q9Y289|SC5A6_HUMAN ----
P13866|SC5A1_HUMAN AYFA 664
Q92911|SC5A5_HUMAN ----
```

Supplementary Fig. 4 Multiple sequence alignment of human SMVT (SC5A6), human SGLT1 (SC5A1), and human NIS (SC5A5) with vSGLT

Multiple sequence alignment of human SMVT (SC5A6; UniProtKB accession number: Q9Y289), human SGLT1 (SC5A1; UniProtKB accession number: P13866), and human NIS (SC5A5; UniProtKB accession number: Q92911) with SGLT of *Vibrio parahaemolyticus* (vSGLT; UniProtKB accession number: P96169) using Clustal Omega. Amino acid residues affected by the variants in SC5A6 are colored in red (R123, Y162, R400, S429).

Supplementary Table 1 Additional clinical features and diagnostic laboratory workup in patients 1-1, 1-2, 1-3, 2-1, and 3-1

		Patient 1-1	Patient 1-2	Patient 1-3	Patient 2-1	Patient 3-1
***SLC5A6* Variant**	NM_021095.4 NP_066918.4	c.1285A>G p.(Ser429Gly) Homozygous	c.1285A>G p.(Ser429Gly) Homozygous	c.1285A>G p.(Ser429Gly) Homozygous	c.280C>T p.(Arg94*) c.485A>G p.(Tyr162Cys)	c.1285A>G p.(Ser429Gly) Homozygous
**Demographics**	Consanguinity	No	No	No	No	No
	Ethnicity	South Asia	South Asia	South Asia	Caucasian	South Asia
	Sex	Female	Female	Male	Female	Female
	Alive	Yes	Yes	Yes	Yes	Yes
**Birth**	Gestational age	37 weeks	37 weeks	37 weeks	40 weeks	37 weeks
	Perinatal complications	No	No	No	No	no
	APGAR	9	9	9	9	9
**Measurements at Birth**	Birth weight (centile) (z)	3100 g (27th) (-0.61)	2900 g (16th) (-0.99)	3500 g (48th) (-0.06)	3210 g (35th) (-0.38)	4000 g (90.3th) (+1.3)
	Birth length (centile) (z)	ND	ND	ND	50.2 cm (64.85th) (0.38)	ND
	OFC birth (centile) (z)	ND	ND	ND	34.5 cm (44.8th) (-0.13)	ND
**Other Features**	Cardiovascular	Normal	Normal	Normal	Dilated cardiomyopathy	Normal
	Hearing	Normal BERA	Normal BERA	ND	ND	ND
	Eye findings	ND	ND	ND	No	Gaze evoked nystagmus
	Craniofacial dysmorphism	No	No	No	No	No
	Behavior abnormalities	No	No	No	No	No
	Skeletal features	Normal	Normal	Normal	Normal	Normal
	Seizures	No	No	No	No	No
	Speech impairment	No	No	No	No	No
	Other findings	Very dry skin; extensive hemangioma involving the entire right upper limb; right inguinal hernia repair at 1.5 y of age	Premature greying of hair; right inguinal hernia repair at 5 y of age	Dry skin	Thin body habitus	None

**Diagnostic Laboratory Workup**	MRI brain	Normal	Normal	Normal	Normal	Normal
	MRI spine	ND	Normal	ND	Enhancement along the nerve roots of the cauda equina	Normal
	Plasma amino acids	Methionine 22.97 µmol/L(75)	Methionine 25.12 µmol/L(75)	Not done	Hydroxyproline 78.1 µmol/L [8.6-45.2], Cystine 32.7 µmol/L [9.8-29.2], Methionine 13.2 µmol/L [13.9-36.5]	ND
	Urine organic acids	Normal	Normal	Not done	Normal	ND
	Urine acylcarnitine profile	Not done	Not done	Not done	Normal	ND
	Plasma acylcarnitine	Normal	Normal	Not done	C0 (unreported), C2 2.06 µmol/L [3.23-10.29], C3 0.13 µmol/L [0.16-0.62]	ND
	Total/free carnitine	Normal	Normal	Not done	Normal	ND
	2-ketoglutarate dehydrogenase activity in heart tissue	Not done	Not done	Not done	Normal	ND
	Pyruvate dehydrogenase complex activity in heart tissue	Not done	Not done	Not done	Normal	ND
	Cardiomyopathy Panel	Not applicable	Not applicable	Not applicable	VOUS in *FHL2* (non-diagnostic)	ND

BERA, brainstem electric response audiometry; m, months; MRI, magnetic resonance imaging; ND, no data; OFC, occipital frontal circumference; VOUS, variant of uncertain significance; wks, weeks; y, years; z, z-score.

Supplementary Table 2 Patient 1-1 nerve conduction studies at age 13 years

Sensory, Motor, F response	L/R	Nerve	Stimulation site	Recording site	Distal latency (motor & F responses only)	Amplitude	Conduction velocity
Sensory	L	Median	Wrist	Digit II		67.2 µV	Not reported
Sensory	R	Median	Wrist	Digit II		58.0 µV	Not reported
Sensory	L	Ulnar	Wrist	Digit V		41.2 µV	Not reported
Sensory	R	Ulnar	Wrist	Digit V		58.0 µV	Not reported
Sensory	L	Radial	Forearm	Wrist		29.1 µV	Not reported
Sensory	R	Radial	Forearm	Wrist		26.8 µV	Not reported
Sensory	L	Lateral antebrachial cutaneous	Elbow	Forearm		22.0 µV	Not reported
Sensory	R	Lateral antebrachial cutaneous	Elbow	Forearm		21.3 µV	Not reported
Sensory	L	Medial antebrachial cutaneous	Elbow	Forearm		21.9 µV	Not reported
Sensory	R	Medial antebrachial cutaneous	Elbow	Forearm		21.1 µV	Not reported
Sensory	L	Femoralis	Inguinal ligament	Thigh		13.7 µV	Not reported
Sensory	R	Femoralis	Inguinal ligament	Thigh		9.2 µV	Not reported
Sensory	L	Superficial fibular (peroneal)	Anterior leg	Anterior ankle		15.2 µV	Not reported
Sensory	R	Superficial fibular (peroneal)	Anterior leg	Anterior ankle		13.2 µV	Not reported
Sensory	L	Sural	Calf	Lateral malleolus		15.2 µV	Not reported
Sensory	R	Sural	Calf	Lateral malleolus		20.6 µV	Not reported
Motor	L	Median	Wrist	APB	3.21 ms	16.6 mV	
			Elbow	APB		13.6 mV	52.5 m/s
			Axilla	APB		13.3 mV	55.9 m/s
			Erb's point	APB		10.2 mV	
Motor	R	Median	Wrist	APB	Absent	Absent	
Motor	L	Ulnar	Wrist	ADM	2.38 ms	12.7 mV	
			Below elbow	ADM		12.4 mV	47.8 m/s
			Above elbow	ADM		12.3 mV	56.5 m/s
			Axilla	ADM		11.9 mV	
			Erb's point	ADM		11.2 mV	
Motor	R	Ulnar	Wrist	ADM	2.52 ms	11.1 mV	
			Below elbow	ADM		9.9 mV	39.7 m/s
			Above elbow	ADM		9.5 mV	46.7 m/s

			Axilla	ADM		9.1 mV	
			Erb's point	ADM		8.7 mV	
Motor	L	Radial	Forearm	EIP	2.21 ms	6.6 mV	
			Antecubital space	EIP		6.0 mV	55.8 m/s
			Spiral groove	EIP		5.3 mV	56.8 m/s
			Erb's point	EIP		5.4 mV	
Motor	R	Radial	Forearm	EIP	2.02 ms	6.0 mV	
			Antecubital space	EIP		6.0 mV	50.0 m/s
			Spiral groove	EIP		5.8 mV	54.6 m/s
			Erb's point	EIP		5.5 mV	
Motor	L	Fibular (peroneal)	Ankle	EDB	3.54 ms	5.3 mV	
			Fibular head	EDB		3.7 mV	41.2 m/s
			Popliteal fossa	EDB		3.5 mV	45.5 m/s
Motor	R	Fibular (peroneal)	Ankle	EDB	4.13 ms	6.9 mV	
			Fibular head	EDB		5.9 mV	41.6 m/s
			Popliteal fossa	EDB		5.6 mV	41.7 m/s
Motor	L	Tibial	Ankle	AH	3.31 ms	6.9 mV	
			Knee	AH		4.4 mV	45.4 m/s
Motor	R	Tibial	Ankle	AH	3.85 ms	8.7 mV	
			Knee	AH		6.4 mV	43.1 m/s
F response	L	Median	Wrist	APB	30.7 ms		
F response	R	Median	Wrist	APB	Absent		
F response	L	Ulnar	Wrist	ADM	31.7 ms		
F response	R	Ulnar	Wrist	ADM	35.7 ms		
F response	L	Fibular (peroneal)	Ankle	EDB	49.8 ms		
F response	R	Fibular (peroneal)	Ankle	EDB	54.6 ms		
F response	L	Tibial	Ankle	AH	51.4 ms		
F response	R	Tibial	Ankle	AH	54.7 ms		

ADM, abductor digiti minimi; AH, abductor halluces; APB, abductor pollicis brevis; EDB, extensor digitorum brevis; EIP, extensor indicis proprius; ms, milliseconds; mV, millivolts; m/s, meters/second; µV, microvolts.

Supplementary Table 3 Patient 1-2 nerve conduction studies at 12 years

Sensory, Motor, F response	L/R	Nerve	Stimulation site	Recording site	Distal latency (motor & F responses only)	Amplitude	Conduction velocity
Sensory	L	Median	Wrist	Digit II		54.72 µV	Not reported
Sensory	R	Median	Wrist	Digit II		54.70 µV	Not reported
Sensory	L	Ulnar	Wrist	Digit V		47.02 µV	Not reported
Sensory	R	Ulnar	Wrist	Digit V		45.66 µV	Not reported
Sensory	L	Radial	Forearm	Wrist		38.69 µV	Not reported
Sensory	R	Radial	Forearm	Wrist		44.59 µV	Not reported
Sensory	L	Lateral antebrachial cutaneous	Elbow	Forearm		16.80 µV	Not reported
Sensory	R	Lateral antebrachial cutaneous	Elbow	Forearm		12.11 µV	Not reported
Sensory	L	Medial antebrachial cutaneous	Elbow	Forearm		27.83 µV	Not reported
Sensory	R	Medial antebrachial cutaneous	Elbow	Forearm		26.20 µV	Not reported
Sensory	L	Femoralis	Inguinal ligament	Thigh		2.15 µV	Not reported
Sensory	R	Femoralis	Inguinal ligament	Thigh		3.02 µV	Not reported
Sensory	L	Saphenous	Medial leg	Ankle		5.92 µV	Not reported
Sensory	R	Saphenous	Medial leg	Ankle		5.21 µV	Not reported
Sensory	L	Superficial fibular (peroneal)	Anterior leg	Anterior ankle		12.60 µV	Not reported
Sensory	R	Superficial fibular (peroneal)	Anterior leg	Anterior ankle		15.25 µV	Not reported
Sensory	L	Sural	Calf	Lateral malleolus		22.21 µV	Not reported
Sensory	R	Sural	Calf	Lateral malleolus		27.77 µV	Not reported
Motor	L	Median	Wrist	APB	2.86 ms	1.412 mV	
			Elbow	APB		0.722 mV	52 m/s
			Axilla	APB		0.638 mV	53 m/s
Motor	R	Median	Wrist	APB	Absent	Absent	
Motor	L	Ulnar	Wrist	ADM	2.55 ms	10.373 mV	
			Below elbow	ADM		9.815 mV	50 m/s
			Above elbow	ADM		9.047 mV	64 m/s
			Axilla	ADM		8.742 mV	
Motor	R	Ulnar	Wrist	ADM	3.13 ms	6.531 mV	
			Below elbow	ADM		5.586 mV	44 m/s
			Above elbow	ADM		5.053 mV	52 m/s
Motor	L	Radial	Forearm	EIP	1.88 ms	6.180 mV	
			Elbow	EIP		5.278 mV	85 m/s
			Spiral groove	EIP		4.791 mV	87 m/s

			Erb’s point	EIP		3.916 mV	
Motor	R	Radial	Forearm	EIP	2.08 ms	6.992 mV	
			Elbow	EIP		6.594 mV	82 m/s
			Spiral groove	EIP		5.226 mV	83 m/s
			Erb’s point	EIP		4.081 mV	
Motor	L	Fibular (peroneal)	Ankle	EDB	2.92 ms	3.351 mV	
			Fibular head	EDB		2.846 mV	52 m/s
			Popliteal fossa	EDB		1.505 mV	53 m/s
Motor	L	Fibular (peroneal)	Fibular head	TA		6.843 mV	
			Popliteal fossa	TA		5.021 mV	
Motor	R	Fibular (peroneal)	Ankle	EDB	3.18 ms	5.267 mV	
			Fibular head	EDB		4.690 mV	44 m/s
			Popliteal fossa	EDB		4.249 mV	56 m/s
Motor	R	Fibular (peroneal)	Fibular head	TA		7.359 mV	
			Popliteal fossa	TA		6.553 mV	
Motor	L	Tibial	Ankle	AH	3.02 ms	6.843 mV	
			Knee	AH		5.021 mV	43 m/s
Motor	R	Tibial	Ankle	AH	4.01 ms	7.974 mV	
			Knee	AH		6.740 mV	42 m/s
F response	L	Median	Wrist	APB	Absent		
F response	R	Median	Wrist	APB	Absent		
F response	L	Ulnar	Wrist	ADM	28.2 ms		
F response	R	Ulnar	Wrist	ADM	32.2 ms		
F response	L	Fibular (peroneal)	Ankle	EDB	46.2 ms		
F response	R	Fibular (peroneal)	Ankle	EDB	47.0 ms		
F response	L	Tibial	Ankle	AH	50.5 ms		
F response	R	Tibial	Ankle	AH	44.4 ms		

ADM, abductor digiti minimi; AH, abductor halluces; APB, abductor pollicis brevis; EDB, extensor digitorum brevis; EIP, extensor indicis proprius; ms, milliseconds; mV, millivolts; m/s, meters/second; TA, tibialis anterior; µV, microvolts. Sensory amplitudes recorded are peak-to-peak.

Supplementary Table 4 Patient 1-3 nerve conduction studies at 7 years

Sensory, Motor, F response	L/R	Nerve	Stimulation site	Recording site	Distal latency (motor & F responses only)	Amplitude	Conduction velocity
Sensory	L	Median	Wrist	Digit II		76.82 µV	Not reported
Sensory	R	Median	Wrist	Digit II		77.24 µV	Not reported
Sensory	L	Ulnar	Wrist	Digit V		89.98 µV	Not reported
Sensory	R	Ulnar	Wrist	Digit V		96.37 µV	Not reported
Sensory	L	Radial	Forearm	Wrist		19.32 µV	Not reported
Sensory	R	Radial	Forearm	Wrist		19.78 µV	Not reported
Sensory	L	Superficial fibular (peroneal)	Anterior leg	Anterior ankle		11.07 µV	Not reported
Sensory	R	Superficial fibular (peroneal)	Anterior leg	Anterior ankle		12.27 µV	Not reported
Sensory	L	Sural	Calf	Lateral malleolus		14.68 µV	Not reported
Sensory	R	Sural	Calf	Lateral malleolus		13.83 µV	Not reported
Motor	L	Median	Wrist	APB	5.26 ms	9.411 mV	
			Elbow	APB		8.450 mV	54 m/s
			Axilla	APB		7.324 mV	59 m/s
Motor	R	Median	Wrist	APB	6.61 ms	0.834 mV	
			Elbow	APB		0.825 mV	41 m/s
			Axilla	APB		0.824 mV	44 m/s
Motor	L	Ulnar	Wrist	ADM	3.44 ms	6.808 mV	
			Below elbow	ADM		5.678 mV	41 m/s
			Above elbow	ADM		5.594 mV	47 m/s
Motor	R	Ulnar	Wrist	ADM	3.28 ms	11.052 mV	
			Below elbow	ADM		7.889 mV	37 m/s
			Above elbow	ADM		6.947 mV	47 m/s
Motor	L	Radial	Forearm	EIP	1.98 ms	9.316 mV	
				EIP		9.249 mV	53 m/s
				EIP		9.104 mV	
Motor	R	Radial	Forearm	EIP	2.24 ms	10.264 mV	
				EIP		10.129 mV	77 m/s
				EIP		8.847 mV	80 m/s
Motor	L	Fibular (peroneal)	Ankle	EDB	4.53 ms	3.333 mV	
			Fibular head	EDB		3.328 mV	41 m/s
			Popliteal fossa	EDB		2.957 mV	57 m/s
Motor	L	Fibular (peroneal)	Fibular head	TA		9.084 mV	
			Popliteal fossa	TA		8.776 mV	77 m/s
Motor	R	Fibular (peroneal)	Ankle	EDB	4.17 ms	3.715 mV	
			Fibular head	EDB		3.285 mV	41 m/s
			Popliteal fossa	EDB		3.216 mV	59 m/s
			Fibular head	TA		9.515 mV	

			Popliteal fossa	TA		9.197 mV	87 m/s
Motor	L	Tibial	Ankle	AH	4.79 ms	10.177 mV	
			Knee	AH		7.063 mV	38 m/s
Motor	R	Tibial	Ankle	AH	3.49 ms	11.910 mV	
			Knee	AH		7.380 mV	38 m/s
F response	L	Median	Wrist	APB	32.6 ms		
F response	R	Median	Wrist	APB	34.7 ms		
F response	L	Ulnar	Wrist	ADM	32.2 ms		
F response	R	Ulnar	Wrist	ADM	32.8 ms		
F response	L	Fibular (peroneal)	Ankle	EDB	44.1 ms		
F response	R	Fibular (peroneal)	Ankle	EDB	50.7 ms		
F response	L	Tibial	Ankle	AH	49.8 ms		
F response	R	Tibial	Ankle	AH	51.5 ms		

ADM, abductor digiti minimi; AH, abductor halluces; APB, abductor pollicis brevis; EDB, extensor digitorum brevis; EIP, extensor indicis proprius; ms, milliseconds; mV, millivolts; m/s, meters/second; µV, microvolts. Sensory amplitudes recorded are peak-to-peak.

Supplementary Table 5 Patient 2-1 nerve conduction studies at 8 years

Sensory, Motor, F response	L/R	Nerve	Stimulation site	Recording site	Distal latency (motor & F responses only)	Amplitude	Conduction velocity
Sensory	L	Median	Wrist	Digit II		28.7 µV	47 m/s
Sensory	L	Sural	Calf	Lateral malleolus		10.3 µV	43 m/s
Motor	L	Fibular (peroneal)	Ankle	EDB	4.32 ms	3.3 mV	
			Fibular head	EDB		3.2 mV	44 m/s
Motor	L	Tibial	Ankle	AH	4.38 ms	4.7 mV	
F response	L	Fibular (peroneal)	Ankle	EDB	40.4 ms		
F response	L	Tibial	Ankle	AH	46.3 ms		

AH, abductor halluces; EDB, extensor digitorum brevis; ms, milliseconds; mV, millivolts; m/s, meters/second; µV, microvolts. Sensory responses recorded are negative peak. The patient did not tolerate further examination.

Supplementary Table 6 Patient 3-1 nerve conduction studies at 13 years

Sensory, Motor, F response	L/R	Nerve	Stimulation site	Recording site	Distal latency (motor & F responses only)	Amplitude	Conduction velocity
Sensory	R	Median	Wrist	Digit II	3.23	50.62 µV	Not reported
Sensory	L	Median	Wrist	Digit II	3.28	55.02 µV	Not reported
Sensory	R	Ulnar	Wrist	Digit V	2.29	40.66 µV	Not reported
Sensory	L	Ulnar	Wrist	Digit V	3.07	43.43 µV	Not reported
Sensory	R	Radial	Forearm	Wrist	1.72	26.31 µV	Not reported
Sensory	L	Radial	Forearm	Wrist	1.72	20.02 µV	Not reported
Sensory	R	Sural	Calf	Ankle	3.33	19.76 µV	Not reported
Sensory	L	Sural	Calf	Ankle	4.06	16.89 µV	Not reported
Sensory	R	Superficial fibular (peroneal)	Lateral leg	Ankle	3.33	11.81 µV	Not reported
Sensory	L	Superficial fibular (peroneal)	Lateral leg	Ankle	3.75	10.26 µV	Not reported
Sensory	R	Lateral antebrachial cutaneous	Elbow	Forearm	-	12.97 µV	Not reported
Sensory	L	Lateral antebrachial cutaneous	Elbow	Forearm	-	15.24 µV	Not reported
Sensory	R	Medial antebrachial cutaneous	Elbow	Forearm	-	11.05 µV	Not reported
Sensory	L	Medial antebrachial cutaneous	Elbow	Forearm	-	15.48 µV	Not reported
Sensory	R	Lateral femoral cutaneous	Anterior inguinal ligament	Thigh	-	1.82 µV	Not reported
Sensory	L	Lateral femoral cutaneous	Anterior inguinal ligament	Thigh	-	1.72 µV	Not reported
Sensory	R	Saphenous	Medial leg	Ankle	-	5.37 µV	Not reported
Sensory	L	Saphenous	Medial leg	Ankle	-	6.78 µV	Not reported
Motor	R	Median	Wrist	APB	3.91 ms	2.034 mV	
			Elbow	APB	8.07 ms	1.880 mV	42 m/s
			Axilla	APB	10.78 ms	1.405 mV	44 m/s
Motor	L	Median	Wrist	APB	4.79 ms	5.536 mV	
			Elbow	APB	9.27 ms	5.064 mV	54 m/s
			Axilla	APB	10.73 ms	4.939 mV	69 m/s
Motor	R	Ulnar	Wrist	ADM	3.25 ms	10.511 mV	

			Below elbow	ADM	8.23 ms	9.957 mV	50 m/s
			Above elbow	ADM	10.00 ms	9.643 mV	50 m/s
Motor	L	Ulnar	Wrist	ADM	2.81 ms	11.501 mV	
			Above elbow	ADM	7.66 ms	10.919 mV	52 m/s
			Below elbow	ADM	9.48 ms	8.936 mV	55 m/s
Motor	R	Fibular (peroneal)	Ankle	EDB	NR	NR	NR
			Fibular head	EDB	NR	NR	NR
			Popliteal fossa	EDB	NR	NR	NR
			Fibular head	TA	6.25	1.435	-
			Popliteal fossa	TA	7.97	1.247	58
Motor	L	Fibular (peroneal)	Ankle	EDB	4.53 ms	2.507 mV	
			Fibular head	EDB		1.340 mV	35 m/s
			Popliteal fossa	EDB		1.063 mV	42 m/s
Motor	R	Tibial	Ankle	AH	3.96 ms	3.852 mV	-
			Popliteal fossa	AH	15.78 ms	2.385 mV	30 m/s
Motor	L	Tibial	Ankle	AH	4.01 ms	2.284 mV	-
			Popliteal fossa	AH	16.09 ms	1.876 mV	29 m/s
Motor	R	Tibial	Popliteal fossa	Gastrocnemius	7.97 ms	2.788 mV	
Motor	L	Tibial	Popliteal fossa	Gastrocnemius	8.85 ms	1.632 mV	
Motor	R	Fibular (peroneal)	Fibular head	TA	6.25 ms	1.435 mV	
			Popliteal fossa	TA		1.247 mV	58 m/s
Motor	L	Fibular (peroneal)	Fibular head	TA	3.44 ms	2.539 mV	
			Popliteal fossa	TA		2.440 mV	58 m/s
Motor	R	Radial	Forearm	EIP	2.08 ms	7.634 mV	
			Elbow	EIP		7.232 mV	66 m/s
			Spiral groove	EIP		6.000 mV	72 m/s
Motor	L	Radial	Forearm	EIP	1.67 ms	8.901 mV	
			Elbow	EIP		7.802 mV	69 m/s
			Spiral groove	EIP		5.805 mV	74 m/s
Motor	R	Axillary	Supraclavicular fossa	Deltoid	3.91 ms	6.739 mV	
Motor	L	Axillary	Supraclavicular fossa	Deltoid	4.06 ms	7.446 mV	
Motor	R	Femoral	Below inguinal ligament	Vastus medialis	3.91 ms	1.034 mV	
Motor	L	Femoral	Below inguinal ligament	Vastus medialis	3.59 ms	1.295 mV	
Motor	R	Musculocutaneous	Supraclavicular fossa	Biceps	4.74 ms	7.176 mV	
Motor	L	Musculocutaneous	Supraclavicular fossa	Biceps	4.95 ms	6.362 mV	
Motor	R	Suprascapular	Erb's point	Supraspinatus	2.03 ms	11.569 mV	
			Erb's point	Infraspinatus	1.93 ms	13.504 mV	
Motor	L	Suprascapular	Erb's point	Supraspinatus	2.03 ms	6.696 mV	
			Erb's point	Infraspinatus	2.45 ms	8.131 mV	
F response	R	Fibular (peroneal)	Ankle	EDB	Absent		
F response	R	Tibial	Ankle	AH	59.3 ms		
F response	L	Fibular (peroneal)	Ankle	EDB	58.2 ms		
F response	L	Tibial	Ankle	AH	61.3 ms		
F response	R	Median	Wrist	APB	34.9 ms		

F response	R	Ulnar	Wrist	ADM	32.3 ms		
F response	L	Median	Wrist	APB	34.1 ms		
F response	L	Ulnar	Wrist	ADM	33.5 ms		

ADM, abductor digiti minimi; AH, abductor halluces; APB, abductor pollicis brevis; EDB, extensor digitorum brevis; EIP, extensor indicis proprius; TA, tibialis anterior; ms, milliseconds; mV, millivolts; m/s, meters/second; µV, microvolts. Sensory amplitudes recorded are peak-to-peak.

Supplementary Table 7 Regions of homozygosity in patient 1-2

#Chr	Begin	End	Size (Mb)	No. of variants	Percentage homozygosity
chr1	65860660	68948280	3.09	33	90.91
chr1	89659134	94341267	4.68	47	89.36
chr1	236212038	237923053	1.71	79	92.41
chr2	25376564	32713706	7.34	140	96.43
chr2	79314094	85662636	6.35	26	92.31
chr5	96129543	97518792	1.39	29	89.66
chr5	139260428	140475980	1.22	50	94
chr5	146775311	147781732	1.01	32	93.75
chr6	13800626	19264210	5.46	30	93.33
chr8	28635303	30699494	2.06	26	88.46
chr11	47204175	48346604	1.14	43	97.67
chr11	49080407	56143198	7.06	53	94.34
chr13	21562482	25264715	3.7	51	88.24
chr17	25917810	27442710	1.52	53	88.68
chr20	13695824	19970705	6.27	46	91.3

A total of 54 Mb homozygous regions (autosomal chromosomes) were seen in patient 1-2. The *SLC5A6* variant c.1285A>G (chr2:27,424,933-T-C) is located in a stretch of 7.34 Mb (highlighted in orange).

Supplementary Table 8 Regions of homozygosity in patient 3-1

#Chr	Begin	End	Size (Mb)	No. of variants	Percentage homozygosity
chr1	204103618	205138990	1.04	34	91.18
chr2	10952790	15758293	4.81	38	89.47
chr2	22865260	28762031	5.9	122	98.36
chr2	75899028	96858914	20.96	113	90.27
chr2	184798504	188211112	3.41	42	92.86
chr3	3194107	8665360	5.47	28	89.29
chr3	108705715	111997731	3.29	37	91.89
chr10	3146180	5014545	1.87	28	89.29
chr10	129245684	130921195	1.68	35	97.14
chr12	121454148	124267703	2.81	111	96.40
chr15	41313064	46968535	5.66	123	95.12
chr15	51507874	53081800	1.57	33	93.94
chr17	40370786	41891437	1.52	60	88.33
chr22	32887150	39966856	7.08	128	97.66

A total of 67.07 Mb homozygous regions (autosomal chromosomes) were seen in patient 3-1. The *SLC5A6* variant c.1285A>G (chr2:27,424,933-T-C) is located in a stretch of 5.9 Mb (highlighted in orange).

Supplementary Table 9 Shared homozygous regions in patients 1-2 and 3-1

#Chr	Begin	End	Size (Mb)
chr2	25376564	28762031	3.39
chr2	79314094	85662636	6.35

The *SLC5A6* variant c.1285A>G (chr2:27424933-T-C) is present in one of the two shared homozygous regions (2:25,376,564-28,762,031) of 3.39 Mb (highlighted in orange).

References:

1. Li H, Durbin R. Fast and accurate short read alignment with Burrows-Wheeler transform. Bioinformatics. 2009;25(14):1754-60.
2. McKenna A, Hanna M, Banks E, Sivachenko A, Cibulskis K, Kernytsky A, et al. The Genome Analysis Toolkit: a MapReduce framework for analyzing next-generation DNA sequencing data. Genome Res. 2010;20(9):1297-303.
3. Wang K, Li M, Hakonarson H. ANNOVAR: functional annotation of genetic variants from high-throughput sequencing data. Nucleic Acids Res. 2010;38(16):e164.
4. Jagadeesh KA, Wenger AM, Berger MJ, Guturu H, Stenson PD, Cooper DN, et al. M-CAP eliminates a majority of variants of uncertain significance in clinical exomes at high sensitivity. Nat Genet. 2016;48(12):1581-6.
5. Ioannidis NM, Rothstein JH, Pejaver V, Middha S, McDonnell SK, Baheti S, et al. REVEL: An Ensemble Method for Predicting the Pathogenicity of Rare Missense Variants. Am J Hum Genet. 2016;99(4):877-85.
6. Kircher M, Witten DM, Jain P, O'Roak BJ, Cooper GM, Shendure J. A general framework for estimating the relative pathogenicity of human genetic variants. Nat Genet. 2014;46(3):310-5.
7. Retterer K, Juusola J, Cho MT, Vitazka P, Millan F, Gibellini F, et al. Clinical application of whole-exome sequencing across clinical indications. Genet Med. 2016;18(7):696-704.
8. Van der Auwera GA, Carneiro MO, Hartl C, Poplin R, Del Angel G, Levy-Moonshine A, et al. From FastQ data to high confidence variant calls: the Genome Analysis Toolkit best practices pipeline. Curr Protoc Bioinformatics. 2013;43:11 0 1- 0 33.
9. Kausthubham N, Shukla A, Gupta N, Bhavani GS, Kulshrestha S, Das Bhowmik A, et al. A data set of variants derived from 1455 clinical and research exomes is efficient in variant prioritization for early-onset monogenic disorders in Indians. Hum Mutat. 2021;42(4):e15-e61.
10. Karczewski KJ, Francioli LC, Tiao G, Cummings BB, Alfoldi J, Wang Q, et al. The mutational constraint spectrum quantified from variation in 141,456 humans. Nature. 2020;581(7809):434-43.
11. Quinodoz M, Peter VG, Bedoni N, Royer Bertrand B, Cisarova K, Salmaninejad A, et al. AutoMap is a high performance homozygosity mapping tool using next-generation sequencing data. Nat Commun. 2021;12(1):518.
